# Identifying Key Genetic Regions for Cell Sheet Morphogenesis on Chromosome 2L Using a *Drosophila* Deficiency Screen in Dorsal Closure

**DOI:** 10.1534/g3.120.401386

**Published:** 2020-09-25

**Authors:** Stephanie M. Fogerson, Richard D. Mortensen, Regan P. Moore, Hellen Y. Chiou, Neel K. Prabhu, Angela H. Wei, Daniel Tsai, Othmane Jadi, Kwabena Andoh-Baidoo, Janice Crawford, Murotiwamambo Mudziviri, Daniel P. Kiehart

**Affiliations:** * Biology Department, Duke University, Durham, NC, 27708; † Department of Pharmacology, University of North Carolina, Chapel Hill, NC, 27599; ‡ Joint Department of Biomedical Engineering, University of North Carolina, Chapel Hill, 27599, USA and North Carolina State University, Raleigh, NC, 27695

**Keywords:** amnioserosa, lateral epidermis, morphogenesis, dorsal closure

## Abstract

Cell sheet morphogenesis is essential for metazoan development and homeostasis of animal form – it contributes to developmental milestones including gastrulation, neural tube closure, heart and palate formation and to tissue maintenance during wound healing. Dorsal closure, a well-characterized stage in *Drosophila* embryogenesis and a model for cell sheet morphogenesis, is a remarkably robust process during which coordination of conserved gene expression patterns and signaling cascades regulate the cellular shape changes and movements. New ‘dorsal closure genes’ continue to be discovered due to advances in imaging and genetics. Here, we extend our previous study of the right arm of the 2^nd^ chromosome to the left arm of the 2^nd^ chromosome using the Bloomington deficiency kit’s set of large deletions, which collectively remove 98.9% of the genes on the left arm of chromosome two (2L) to identify ‘dorsal closure deficiencies’. We successfully screened 87.2% of the genes and identified diverse dorsal closure defects in embryos homozygous for 49 deficiencies, 27 of which delete no known dorsal closure gene. These homozygous deficiencies cause defects in cell shape, canthus formation and tissue dynamics. Within these deficiencies, we have identified *pimples*, *odd-skipped*, *paired*, and *sloppy-paired 1* as dorsal closure genes on 2L that affect lateral epidermal cells. We will continue to identify novel ‘dorsal closure genes’ with further analysis. These forward genetic screens are expected to identify new processes and pathways that contribute to closure and links between pathways and structures already known to coordinate various aspects of closure.

Cell sheet morphogenesis is responsible for essential developmental hallmarks such as gastrulation, neural tube closure, heart and palate formation as well as tissue maintenance through wound healing (Knust 1997; Keller *et al.* 2003; Martin and Parkhurst 2004; Ray and Niswander 2016) . Morphogenesis is a sequence of cell shape changes and movements modulated by changes in cytoskeletal structure and cell-cell and cell-matrix adhesion that are complex. A comprehensive list of all of the molecular players that participate in morphogenesis is necessary for understanding how gene regulatory networks, signaling pathways and their protein effectors initiate, regulate and drive morphogenesis.

*Drosophila* dorsal closure occurs midway through embryogenesis and provides a well-characterized and tractable model for epithelial sheet morphogenesis. During closure, two lateral epidermal sheets extend toward the dorsal midline of the embryo to cover a hole filled with a transient epithelial tissue, the amnioserosa (Figure 1, here and in most figures images in panels are augmented with supplemental movies). Both the lateral epidermis and amnioserosa provide forces that contribute to morphogenesis. The amnioserosa cells pulsate (oscillate) and eventually contract, ingress, and apoptose, pulling the lateral epidermis toward the dorsal midline. Simultaneously, the dorsal-most cells of the lateral epidermis lengthen along the dorsal-ventral, circumferential axis. Near the border between the dorsal-most epithelial (DME) cells and the peripheral amnioserosa (PAS) cells, continuous supracellular, actomyosin rich purse-strings (or cables) are formed. The purse-strings also generate forces that help pull the two flanking sheets of lateral epidermis together. Closure is a remarkably robust, resilient, and redundant process. Numerous components of conserved gene regulatory networks and signaling cascades are required to regulate the cellular machines that drive closure (Harden 2002; Jacinto *et al.* 2002b; Hayes and Solon 2017; Kiehart *et al.* 2017). Dorsal closure often proceeds to completion when one of the force-producing tissues is completely removed or compromised, either by laser microsurgery or genetic manipulations (Hutson *et al.* 2003; Muliyil and Narasimha 2014; Wells *et al.* 2014).

**Figure 1:**
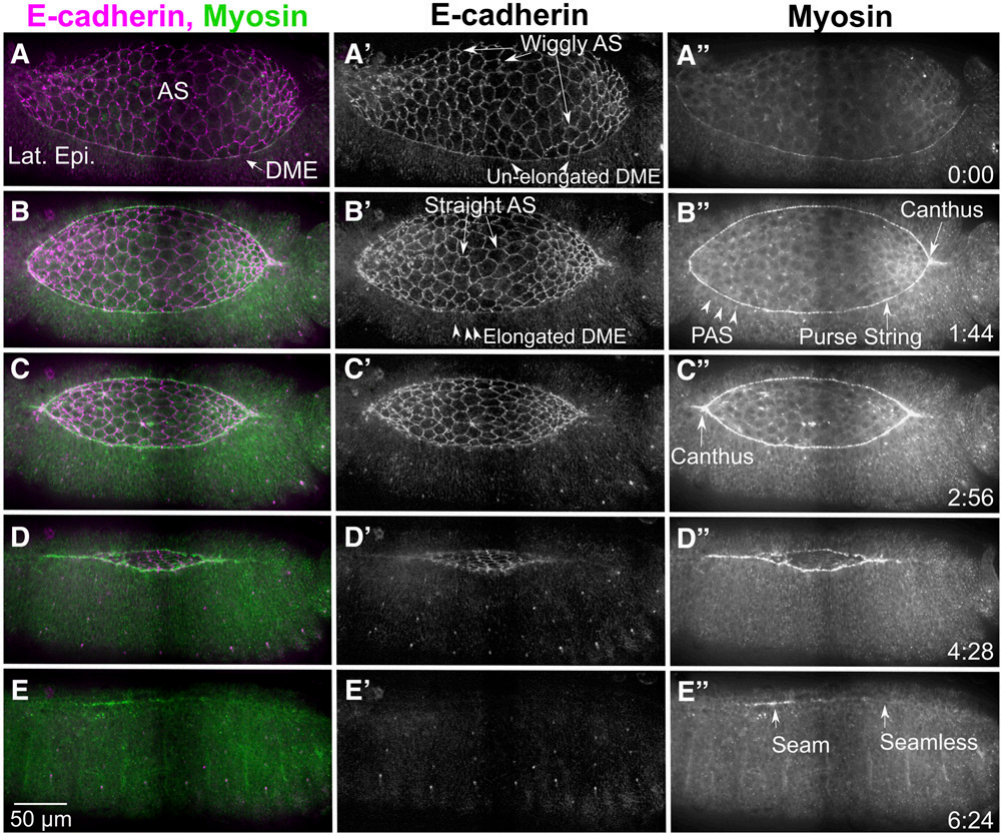
Dorsal closure progression from pre-canthus formation to a seamed epithelium. The cellular morphologies and cytoskeletal dynamics during dorsal closure are shown here by endogenously labeling cadherin at the adherens junctions (Ecad-Tomato, A’-E’) and myosin (myosin heavy chain-GFP exon trap, A”-E”) in stills taken from a stitched confocal time-lapse sequence. Prior to dorsal closure, the ends of the dorsal opening are blunt or rounded, the dorsal most epithelial (DME) cells are isotropic (unstretched), the amnioserosa have wiggly cell junctions and myosin is weakly localized to the boundary between the amnioserosa (AS) and lateral epidermis (Lat. Epi., A-A”) where the purse string will form. At the onset of dorsal closure, a canthus forms at the posterior end of the dorsal opening as zipping begins while the anterior end remains rounded (B-B”). The DME cells begin to elongate along their circumferential, dorsal-ventral axis (B’), while the peripheral amnioserosa (PAS) cells tuck under the DME cells (B”). The junctions of the bulk amnioserosa cells straighten and myosin bars accumulate at the purse string (B-B”). The anterior canthus soon forms (C-C”) and the lateral epidermal sheets zip together from both ends causing the dorsal opening to decrease in height (along the dorsal-ventral axis) and width (anterior-posterior axis, C-D”). Once dorsal closure completes, there is a seamed, and later seamless, epithelium (E-E”). Anterior is to the left, posterior to the right in all panels. Time is in hr:min. The scale bar in E applies to all panels (50 µm). Stills are from Supplemental Movie (Suppl Mov) 1, available on figshare.

Genetic screens uncovered a substantial fraction of the ∼140 known ‘dorsal closure genes’ – genes, that when mutated cause defects in closure. Most of these screens used the terminal “dorsal open” phenotype in the cuticle as a readout for dorsal closure defects, thus only the most severe dorsal closure genes were uncovered (Nüsslein-Volhard and Wieschaus 1980; Jurgens *et al.* 1984; Nüsslein-Volhard *et al.* 1984; Wieschaus *et al.* 1984). In 2011, an RNAi screen for candidate dorsal closure genes used a live imaging approach that yielded six new dorsal closure genes and revealed that some dorsal closure gene knock-downs cause major defects in tissue morphology and tissue dynamics, but still complete dorsal closure (Jankovics *et al.* 2011). Moreover, new dorsal closure genes are continually discovered by candidate gene approaches or serendipity.

Recently, we conducted a forward genetic screen designed to uncover all zygotically expressed genes on the right arm of Chromosome 2 (2R) that are required for the normal kinematics and dynamics of dorsal closure (Mortensen *et al.* 2018). Chromosome arm 2R constitutes ∼1/5^th^ of the fly genome. We utilized the Bloomington Stock Center’s deficiency (Df) kit for 2R, which includes a set of 98 large deletions, that collectively remove 98.5% of the genes (Cook *et al.* 2012). In two crosses, we identified homozygous Df embryos and imaged them through the duration of closure (Figure 2). We found dorsal closure defects in 47 Dfs of the 92 Dfs screened: embryos homozygous for the Dfs displayed a variety of phenotypes which collectively affected all tissues and processes that contribute to closure (so called dorsal closure Dfs). Thus far, the 2R screen identified 4 new “dorsal closure-” or “pre-dorsal closure- genes” that caused the observed, dorsal closure Df phenotypes. Three of these genes have distinct lateral epidermal phenotypes. Two have large lateral epidermal cells that are otherwise normally shaped – *three rows* and *tumbleweed*. The third, *even skipped* (*eve*), similarly has large lateral epidermal cells but they are also isotropic – not anisotropic in shape like their wild-type counterparts (Figure 1). The fourth new dorsal closure gene we identified, *jelly belly*, caused a rounded dorsal opening. The results of the Df screen indicated that many key genes that contribute to the kinematics and dynamics of closure have yet to be identified. In sum, efficiently screening for complete loss of zygotic gene function in an unbiased approach has uncovered gene and gene regions that were unexpected candidates for dorsal closure defects.

**Figure 2:**
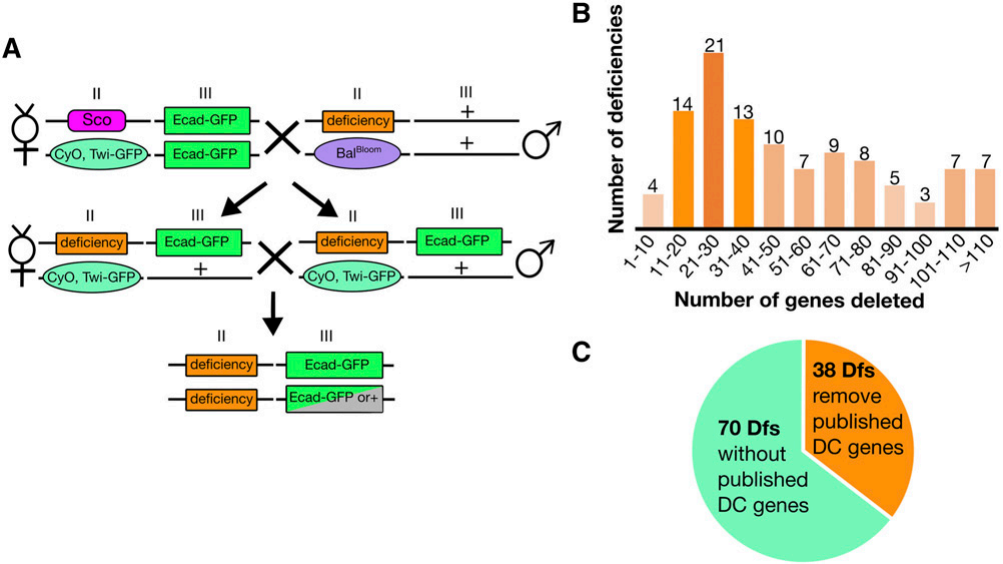
Crossing scheme for live imaging deficiencies. Following two crosses, embryos homozygous for a Df can be selected by the lack of Twi-GFP and imaged with Ecad-GFP (A). The 2^nd^ and 3^rd^ chromosomes are indicated by Roman numerals II and III, respectively. The balancer from the Df stock is indicated by Bal^Bloom^. 2L Dfs remove as few as five genes and as many as 217 genes, the distribution of 2L Dfs by number of genes removed are shown, where the lightest orange represents 1-5 Dfs and the darkest orange represents 20 or more Dfs (B). Thirty-eight of the 108 2L Dfs remove genes documented to cause dorsal closure defects when mutated, while 70 Dfs do not (C).

Here, we extend our dorsal closure Df study to the left arm of the 2^nd^ chromosome (2L) using the Bloomington 2L deficiency kit along with six additional 2L deficiencies. We imaged embryos homozygous for 99 out of the 108 2L Dfs, thereby analyzing most of the genes on 2L for their contribution to closure. Fifty-five percent of the dorsal closure Dfs we analyzed do not remove a known dorsal closure gene and we have begun to identify the genes responsible for the dorsal closure defects. We have also added a new Df dorsal closure phenotype category – exacerbated asymmetric zipping – embryos homozygous for four 2L Dfs display this phenotype. The Df kit has allowed us to screen the second chromosome to near saturation for novel dorsal closure genes and pathways and has uncovered 62 genomic regions that contain one or more novel dorsal closure gene(s).

## Materials and Methods

### Drosophila stocks

All stocks used in the 2L Deficiency (Df) screen are from Bloomington Stock Center (Bloomington, IN). The 2L Df kit stocks were assigned a screen name (*Df(2L)n*) based on chromosomal position, where the Df most distal to the centromere is named *Df(2L)01* and the Df most proximal is *Df(2L)102*. *Df(2L)41^A^* (BL9186), *Df(2L)41^B^* (BL7501), *Df(2L)86^A^* (BL7833), *Df(2L)95^A^* (BL7525), *Df(2L)96^A^* (BL23158), and *Df(2L)99^A^* (BL9340) were added to the screen to provide further coverage of the original 2L Df kit for reasons described below. These Dfs are named with the same 2L Df number as the kit Df it spans and are distinguished from the kit Df with a superscript, uppercase letter. Thus, the nomenclature indicates which genomic region the new Df is supplementing. If multiple Dfs were added for one kit Df then the most distal is superscript A, followed by B, etc. The Bloomington stock numbers and FlyBase nomenclature are included in Appendix A next to the *Df(2L)n* designation for each Df. All overlapping deficiencies and mutant stocks used are listed with the corresponding Bloomington stock numbers in Appendix B. Control embryos for the 2L Df screen are *w^1118^* with a transgene on the third chromosome that ubiquitously expresses *D*E-cadherin-GFP (Ecad-GFP, labeling cell junctions, Oda and Tsukita 2001). The fluorescently marked balancer used to select homozygous embryos (*Twist-GFP*, *CyO* (*TGC*)) is from the *wg*^*Gla-1*^* / CyO*,*twist-Gal4*::*UAS2xEGFP* stock (BL6662, Halfon *et al.* 2002). The control embryo displayed in Figure 1 is labeled with fluorescent *D*E-cadherin and myosin and was generated by recombining *DE-cadherin-mTomato* from Huang *et al.* (2009) with *zipper-GFP* from Buszczak *et al.* (2007).

### Crossing scheme

We used two crosses to generate homozygous Df embryos in an “imaging line” background (Figure 2, Mortensen *et al.* 2018). In cross 1) virgin females of *sna*^*Sco*^ / *TGC*; *Ecad-GFP* were mated to *Df(2L)n* / *Balancer^Bloomington^* (*Bal^Bloom^*) males. Cross 2) was an *inter se* cross between siblings of Cross 1: ∼20 virgin females *Df(2L)n* / *TGC*; *Ecad-GFP* / + progeny to ∼10 males *Df(2L)n* / *TGC*; *Ecad-GFP* / + . Cross 2 was set up in an embryo collection cage and embryos were collected for live imaging. The Ecad-GFP labels the junctional belts or adherens junctions, providing a continuous line around all cells in dorsal closure tissues, thereby allowing us to visualize and analyze cell morphology and kinematics of closure. The *Ecad-GFP* transgene is an overexpression of functional *D*E-cadherin and our previous 2R pilot screen documented cases where Dfs covering cadherin and cadherin-based adhesion loci were rescued by the imaging line. Otherwise, no other significant artifacts were observed and the clear labeling of cell junctions is ideal for the analysis of cell shape changes that characterize dorsal closure defects. All crosses were maintained on standard cornmeal/molasses fly food or in embryo collection cages on grape juice agar at 25°.

### Embryo collection and imaging preparation

Embryos were usually collected for 4 hr at 25° and aged at 16° for ∼24 hr. In some cases, dorsal closure stage, dechorionated embryos were hand selected from overnight collections. Chorions were removed by soaking in 50% bleach for 1.25 min, then washed extensively with deionized H_2_O. Embryos were manually sorted for early dorsal closure stage by morphological hallmarks such as the heart shaped opening on the dorsal surface using reflected light or fluorescence and a dissecting microscope. Then *Df(2L)n* homozygous embryos were selected under GFP fluorescence using the Zeiss Discovery V12 SteREO dissecting microscope (Carl Zeiss, Thornwood, NY), selecting away from the fluorescently marked balancer. Ten to twelve *Df(2L)n* / *Df(2L)n*; *Ecad-GFP* / *Ecad-GFP* or *Df(2L)n* / *Df(2L)n*; *Ecad-GFP* / + embryos were lined up along with control embryos (+ / +; *Ecad-GFP*) collected in parallel. Embryos were oriented on a grape plate, then picked up on a coverslip coated with a thin layer of embryo glue (Kiehart *et al.* 2000a). The embryos were covered with halocarbon oil and the coverslip was mounted on a teflon windowed chamber (Kiehart *et al.* 2006).

### Imaging

In order to analyze dorsal closure in the Df homozygotes, we used a Yokogawa CSU-10 spinning disk confocal head (Perkin Elmer) attached to either a Zeiss Axiovert 200M microscope with a Hamamatsu Orca-ER CCD camera using Metamorph software (Molecular Devices, San Hose, CA) or a Zeiss Axio Imager.M2m microscope with a Hamamatsu EM-CCD (C9100-12) camera using Micro-manager software (Open Imaging, San Francisco, CA). Three to four well oriented, early dorsal closure stage embryos with a dorsal opening height, measured along the dorsal-ventral axis, of approximately 75-100 µm for each Df and 2-3 control embryos were selected for imaging. Note that this is the height of the dorsal opening as measured from the micrographs. Other publications have referred to this axis as the width of the dorsal opening as it is the short axis of the embryo. For consistency with previous publications from the lab, we refer to the dorsal-ventral axis of the dorsal opening as the height. A Zeiss multi-immersion 40X, 0.9 N.A. objective, Zeiss oil-immersion 40X, 1.3 N.A. objective, or a Zeiss water-immersion 40X, 1.2 N.A. objective was used. A 12-16 stack z series with a 1 µm step size was acquired every 2 min until the completion of dorsal closure or clear failure of closure in Dfs or mutant embryos (dorsal closure takes approximately 3-4 hr to complete in wild-type embryos). All images were acquired at a 500 ms exposure time with a gain of 120 and 2x2 binning on the Zeiss Axiovert 200M or 1x1 binning on the Zeiss Axio Imager.M2m. At least six embryos acquired on two different days were analyzed for each Df stock.

### Image processing and analysis

Images were processed using Metamorph (Molecular Devices, San Hose, CA), Micro-manager software (Open Imaging, San Francisco, CA), and Fiji/ImageJ2 software (Schindelin *et al.* 2012). Maximum intensity z-projections were generated for all embryos imaged. When appropriate, planes imaged too deep (thereby including yolk autofluorescence) were omitted before compiling the projections as the autofluorescence of the yolk made it difficult to score the cell shapes of the amnioserosa and lateral epidermis (Sokolow *et al.* 2012). Background subtraction using a rolling ball radius of 150-200 pixels was used to improve fluorescence signal to noise. Additionally, to better define cell shapes, especially in images highlighting the lateral epidermis, the unsharp mask filter with a radius of 1 pixel and mask weight of 0.30 was used. Dfs were explicitly compared to control embryos imaged simultaneously to control for specimen preparation artifacts, microscope or temperature variations during experimental run. They were also compared to all of the control embryos imaged across the duration of the 2R and 2L Df screens to account for variability within dorsal closure of Ecad-GFP embryos. We qualitatively assessed the cell shapes and the kinematics of the amnioserosa cells and lateral epidermis cells for each homozygous Df and scored them based on severity of phenotype, the tissue(s) affected and the observed penetrance (fraction of embryos showing the phenotype). We stipulated that a 50% or greater penetrance was required for a phenotype to be scored as having a mid-severe to strong dorsal closure defect. Often when a mid-severity or strong phenotype had a penetrance between 25–50% the number of embryos screened was increased to ensure a genomic region of interest was not missed due to small sample size. The analysis was done in a blind fashion such that it was unknown to the investigator whether the Df being analyzed deleted a known dorsal closure gene. This was designed to prevent bias in the classification of the phenotype. Once analysis was complete for all 2L Dfs, the phenotypes of embryos homozygous for Dfs that removed known dorsal closure genes were compared to the published dorsal closure phenotype in order to assess whether the known dorsal closure gene could explain the observed phenotype of the Df.

### Amnioserosa oscillation analysis

Dfs suspected of having oscillation defects in the amnioserosa at the low time resolution of two minutes were reimaged at 30-32 sec intervals. The area of 30 to 40 central, non-neighboring amnioserosa cells for each Df was measured for 30 frames (thus following a cell for 15-16 min) using the polygon selection tool in FIJI/ImageJ. MATLAB was then used to calculate the amplitude and period of oscillation as previously described in Blanchard *et al.* (2010) and Moore (2018). A two sample Kolmogorov-Smirnov test was used to determine if there was a difference in amplitude and/or period between the Df and controls. P-values less than 0.05 are reported as significant.

### Data availability

All renewable reagents not already publicly available will be shared upon request. The authors confirm that all data necessary for verifying the conclusions in this article are fully represented by the figures and tables provided. Supplemental materials include: Appendix A – a detailed summary of all Dfs analyzed in this study including total number of embryos imaged/analyzed, dorsal closure phenotype severity, tissues and processes affected, and comparison to known dorsal closure gene(s) if they were removed by a Df, Appendix B – all additional stocks used in the study not included in the Bloomington 2L Df kit, and supplemental movies for all stills of figures with file names including the figure number and panel as well as the genotype. Supplemental material available at figshare: https://doi.org/10.25387/g3.12895142.

## Results and Discussion

### Screening the 2L deficiency kit

Through two crosses, we are able to unambiguously identify and live image embryos homozygous for each 2L Df kit stock by introducing a fluorescently marked balancer chromosome and one or two copies of *DE-cadherin-GFP* (*Ecad-GFP*) for imaging cell shapes. By selecting away from the bright GFP signal in the mesoderm which marks the balancer chromosome, we clearly identified embryos that are homozygous for the Df (Figure 2A). The Ecad-GFP labels the adherens junctions (junctional belts) of the amnioserosa and lateral epidermis, thereby providing a clear outline of cell shapes that allows for the tracking of cell morphology and dynamic changes at the level of the adherens junctions throughout dorsal closure (Oda and Tsukita 2001). The 2L Df kit’s 102 stocks provide 98.9% coverage of the 2,765 euchromatic genes on the left arm of the second chromosome. The Dfs in the kit remove 5 to 217 genes with a median of 42 genes, allowing for quick screening across nearly the entire 2L chromosome (Figure 2B). We added six additional Dfs, *Df(2L)41^A^*, *Df(2L)41^B^*, *Df(2L)86^A^*, *Df(2L)95^A^*, *Df(2L)96^A^*, and *Df(2L)99^A^*, to provide further coverage (see below for Dfs unable to image and tissue failure prior to dorsal closure). In total we were able to image 99 of the 108 Df stocks and screened 87.2% of genes on 2L for dorsal closure phenotypes. Thirty-eight Dfs remove genes previously published to be involved in dorsal closure (“known dorsal closure gene(s)”, Figure 2C). For analysis purposes the Dfs were scored blindly, where neither the imager nor analyzer knew whether a Df removed a known dorsal closure gene. *In vivo* imaging of all 99 Df stocks were qualitatively evaluated for defects in tissue morphology and kinematics from the onset through the completion of dorsal closure and were compared to control dorsal closure embryos. At least six embryos that were homozygous for the Df were analyzed for each 2L Df stock. All embryos were scored and phenotypes that had an expressive phenotype with a penetrance of 50% or more were counted. It is important to note that the aging of embryos at the low temperature of 16° for 24 hr prior to imaging may suppress or enhance dorsal closure defects of a Df; however, we have only observed one instance where the Df dorsal closure phenotype severity changed based on the temperature at which the embryos were aged.

Dfs were ranked into six categories based on the severity, expressivity and penetrance of the dorsal closure phenotype they caused (as described for our 2R Df screen, see Appendix A). Ten Dfs caused a very severe and penetrant dorsal closure phenotype which affected the morphology and/or kinematics of closure to the extent that closure failed to proceed to completion. These Dfs were classified as “strong and fail”. Eight Dfs caused a “strong but closes” phenotype whereby one or more of the processes necessary for dorsal closure were obviously altered to the point that makes it surprising that a majority of the affected embryos completed closure. In many of these cases, noticeable defects, such as scarring at the seam characterized the formed, dorsal epithelium. Thirty-one of the 2L Dfs had a “mid-severity” dorsal closure phenotype. This class was the most prevalent and included Dfs which caused an expressive and penetrant phenotype (greater than 50%) that is less severe than the strong dorsal closure phenotypes but were clearly distinguishable from control embryos. Twenty-two Dfs caused a “weak” dorsal closure phenotype. Embryos homozygous for these Dfs had subtle differences from control embryos but these were either not penetrant (less than 50%) or not unambiguously expressive – *i.e.*, distinguishable from control embryos. Embryos homozygous for 22 of the 2L Dfs were indistinguishable from control embryos, had no dorsal closure phenotype, and were therefore classified as “none”. Six Dfs had severe tissue failure prior to dorsal closure – the dorsal closure tissues were absent or unrecognizable at the dorsal closure stage, these Dfs were classified as “fails prior to closure” (see below “Tissue failure prior to dorsal closure” for more information). These embryos were more severe than the pre-dorsal closure phenotypes described previously in that they failed to undergo any form of closure (Mortensen *et al.* 2018).

The 49 Dfs that caused a strong or mid-severity dorsal closure phenotype, 27 of which do not remove a known dorsal closure gene, were further analyzed and classified based on the tissues and processes disrupted in dorsal closure. Eighteen of the 49 “dorsal closure” Dfs start dorsal closure with aberrant cell shapes and tissue organization; therefore, the gene(s) responsible for the defect are functioning prior to dorsal closure. Nonetheless, these alterations in cell shape and tissue organization impact the process of dorsal closure and can be highly instructive as to the key requirements for dorsal closure to proceed. Moreover, they demonstrate the robust and resilient mechanisms of morphogenesis. Therefore we have included these “pre-dorsal closure” Dfs in our analysis of the 2L Df kit for genes affecting dorsal closure. As in the screen of 2R, all 2L dorsal closure and pre-dorsal closure Dfs were grouped based on how they affected the amnioserosa, the lateral epidermis, zipping and canthus formation, and the interface between the PAS and the DME cells. Several of the Dfs caused multiple phenotypes that affected more than one tissue and/or process and were grouped accordingly. A summary for each Df appears in Appendix A. When two or more phenotypes are uncovered by a Df, the Df is included in the category for each phenotype observed. It is important to note that primary defects in one tissue or process can lead to additional defects in the same tissue or neighboring tissue or process. In such cases, we could not determine with complete certainty whether one defect was the cause for the other.

An overview of how various dorsal closure tissues and processes were perturbed follows. Amnioserosa defects included irregular cell shapes, loss of tissue integrity where holes developed in and between cells, and/or changes in ingression. Lateral epidermal phenotypes included increased cell size, changes in morphology *i.e.*, isotropic (isodiametric) *vs*. anisotropic cell shapes, and disorganization of the cell sheet. Defects in zipping and canthus structure caused scarring, oblong dorsal opening shapes, missing or malformed canthi, and exacerbated asymmetry in zipping. Phenotypes at the interface between the PAS cells and DME cells of the lateral epidermis manifested as jagged/wavy or rounded dorsal openings and/or tearing between the PAS and DME cells. Defects at the interface between the PAS and DME may have been due to defects in one or both cell types as was likely the case in tearing between the PAS and DME cells. Alternatively, defects in dorsal opening shapes could have been due to more global defects in the amnioserosa and/or lateral epidermis. Understanding the specific ways in which tissues and processes are disrupted by a Df provides insight into the individual, mutable mechanisms that contribute to dorsal closure, how disruption affects the otherwise normal tissues and processes of closure, and what aspects are absolutely necessary for dorsal closure to complete. This analysis of Df phenotypes also informs how we prioritize analysis of the genes whose loss might account for the observed dorsal closure Df phenotypes.

### Amnioserosa phenotypes (29 Dfs)

The amnioserosa is a thin sheet of squamous epithelium covering the dorsal hole. The amnioserosa cells pulsate or oscillate, contracting and relaxing their cell area (measured at the level of the adherens junctions). Throughout closure, a subset of cells contract, then ingress, primarily at the canthi and near the purse-strings, giving these cells the name “marginal cells” (Gorfinkiel *et al.* 2009; Blanchard *et al.* 2010; Sokolow *et al.* 2012). Amnioserosa cells ingress without disrupting tissue integrity. Together the contractions and ingressions cause a reduction in amnioserosa area that serves to pull the flanking lateral epidermis toward the dorsal midline. In wild-type embryos, the amnioserosa is estimated to contribute approximately 75–78% of the force toward normal dorsal closure (Hutson *et al.* 2003; Peralta *et al.* 2007). Understanding which genes are important for amnioserosa tissue morphology, integrity, and dynamics is essential for understanding the mechanisms of dorsal closure. Moreover, these findings can apply to other morphogenetic processes that require tissue integrity and junctional remodeling such as gastrulation and neural tube closure in vertebrates (Parameswaran and Tam 1995; Christodoulou and Skourides 2015).

**Figure 3:**
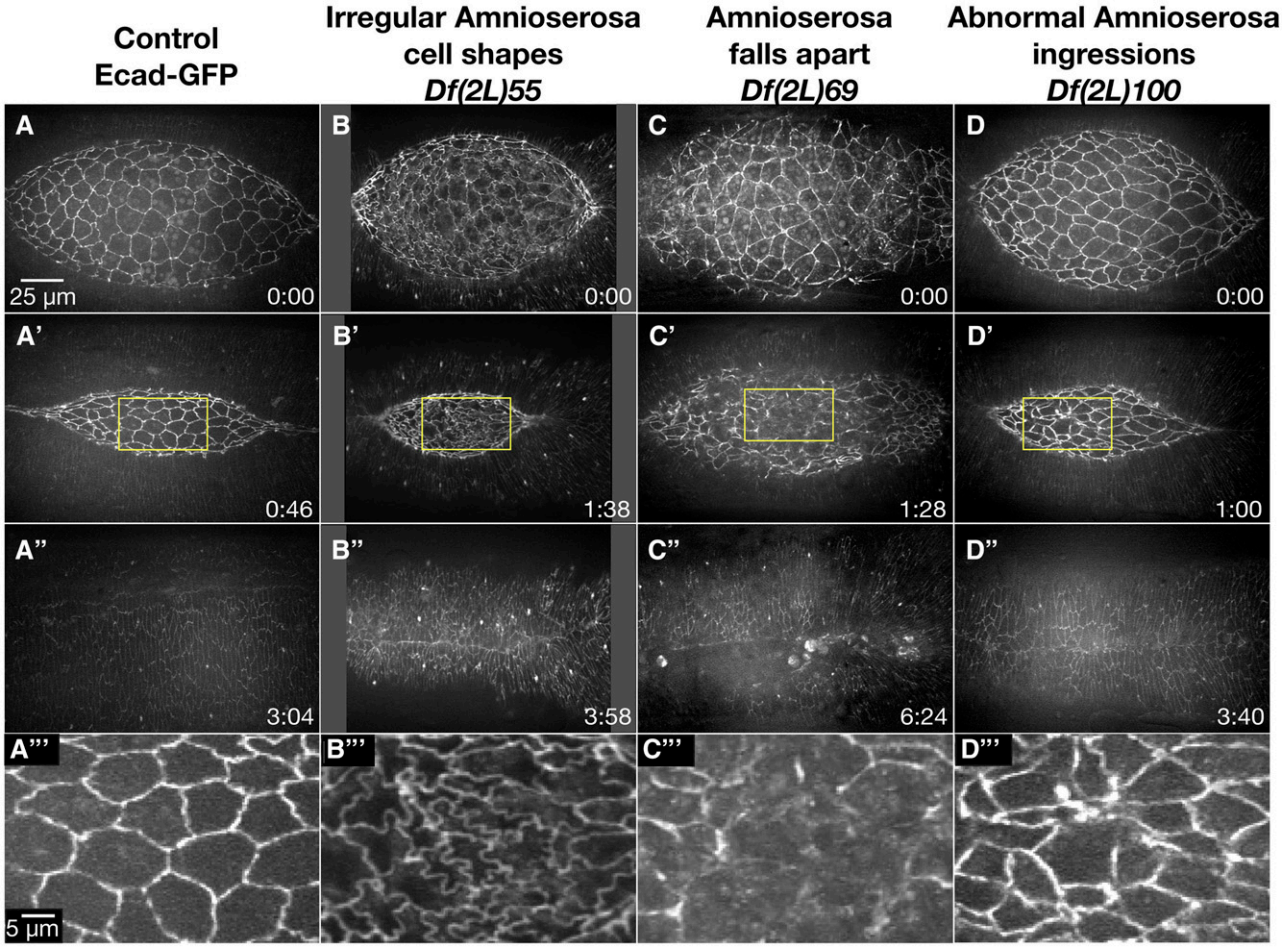
Amnioserosa phenotypes observed in homozygous 2L Df embryos. Stills from a time-lapse sequence of Ecad-GFP labeled control embryos (A-A’’’) and examples of homozygous Df embryos that display amnioserosa phenotypes. *Df(2L)55* embryos have irregular amnioserosa cell shapes (B-B’’’). In *Df(2L)69* embryos, the amnioserosa cell sheet falls apart (C-C’’’). *Df(2L)100* embryos show abnormal amnioserosa cell ingressions (D-D’’’). The yellow boxed areas in A’, B’, C’, and D’ are magnified in A’’’, B’’’, C’’’, and D’’’. Anterior is to the left, posterior to the right. Time is in hr:min, time 0:00 is at the start of the experimental run when the height of the dorsal opening was between 75-100 µm. The scale bar in A applies to panels A-D” and the scale bar in A’’’ applies to panels A’’’-D’’’. Stills are from Suppl Movies 2-5.

Embryos homozygous for 29 different 2L Dfs caused amnioserosa defects during dorsal closure (Table 1). We separated the amnioserosa phenotypes into three qualitative categories: irregular amnioserosa cell shapes, amnioserosa falls apart and irregular amnioserosa cell ingressions. Some Dfs caused phenotypes in multiple amnioserosa categories – such complex phenotypes may indeed be linked to one another. Examples for the three phenotype categories are presented in Figure 3.

**Table 1:** Deficiencies that cause amnioserosa phenotypes (29 total)

Amnioserosa Phenotype	Number of Dfs	Screen Name Df(2L)n
Irregular amnioserosa cell shapes	**16**	12, 35, 38, 43, 45, 48, 51, 54, 55, 56, 61, 76, 84, 93, 94, 98
Amnioserosa falls apart	**14**	05, 07, 14, 22, 29, 48, 54, 61, 68, 69, 76, 92, 93, 94
Abnormal amnioserosa ingressions	**13**	08, 12, 17, 38, 42, 43, 45, 51, 52, 68, 92, 94, 100

The table lists the three classes of amnioserosa phenotypes which were observed in embryos homozygous for Dfs, the number of Dfs that caused the phenotypes and the Dfs that caused them. Note that Dfs are denoted by chromosomal position (see Material and Methods), the corresponding Bloomington stock numbers and the FlyBase nomenclature can be found in Appendix A. Some Dfs caused phenotypes in more than one category.

Oscillations are an integral part of cell shape changes during dorsal closure, but the two minute time interval used for imaging during the screen is not fast enough to unambiguously characterize their period or amplitude (Mortensen *et al.* 2018). For this reason, oscillation defects are not included as a category for amnioserosa phenotypes. Nevertheless, we do report five Dfs in Appendix A with confirmed oscillation defects in amplitude (*Df(2L)34*, *47*, *55*, *61* and *97*) and period (*Df(2L)34* and *61*). These Dfs were suspected to have oscillation defects based on the low time resolution time-lapse records of the initial screen and then confirmed with re-imaging at faster acquisitions (30-32 sec) that were sensitive enough to detect whether an oscillation defect was present.

#### Irregular amnioserosa cell shapes:

Amnioserosa cells are nearly isotropic (*i.e.*, isodiametric) with “wiggly” cell borders in early dorsal closure. Cell borders straighten out in mid dorsal closure and cells become oriented more anisotropically, elongating along the anterior/posterior axis (Gorfinkiel *et al.* 2009; Blanchard *et al.* 2010; Mateus *et al.* 2011).

Sixteen 2L Dfs had irregularly shaped amnioserosa cells (Table 1). *Df(2L)55*, a mid-severity dorsal closure Df, provides an example of amnioserosa cell shapes that remained wiggly throughout dorsal closure (Figure 3B-B’’’). The rate of dorsal closure was slower than controls and may be due to a reduction in ingressions – cells appeared to be compressed by neighboring cells rather than gradually decreasing their cell area and often did not fully ingress. We speculate that the resolution of the wiggly borders into straight boundaries may be necessary for ingression to occur efficiently or conversely, that ingression is a key mechanism by which wiggly borders are resolved into straight ones. Similarly, we found that the amplitude of oscillations in amnioserosa cells of embryos homozygous for *Df(2L)55* was reduced and may have contributed to the slowed rate of closure as the pulsatile amnioserosa behavior is thought to contribute to the dorsal-ward force (reviewed in Gorfinkiel 2016). It has been previously shown that mutations that cause wiggly amnioserosa cell shapes through the reduction of endocytosis also cause a delay in amnioserosa apical constriction and a reduced rate of closure (Mateus *et al.* 2011). This suggests that the resolution of wiggly cell boundaries of the early amnioserosa to straight borders in mid-dorsal closure is important for proper oscillatory morphogenesis. Once the gene responsible for the wiggly amnioserosa cell boundaries is identified it will be important to do a thorough analysis of the changes in amnioserosa morphogenesis. Additionally, 50% of the *Df(2L)55* homozygous embryos imaged also had severe puckering at the end of dorsal closure. It is not clear whether the amnioserosa phenotypes contributed to this puckering or if the puckering was an independent phenotype – analysis of overlapping sub-Dfs to narrow down the genomic region responsible for these dorsal closure phenotypes may provide further insight into the dependence of the two phenotypes on one another.

The majority of the remaining 15 Dfs with irregular amnioserosa cell shapes either maintained wiggly borders into late stages of dorsal closure or the amnioserosa was disorganized with small, non-ingressing cells intermixed with larger cells, comparable in size to wild-type.

#### Amnioserosa falls apart:

Tissue adhesion is essential for maintaining the integrity of an epithelium and the integrity of the amnioserosa is also important for coordinating the dynamic morphogenetic movements that contribute to dorsal closure in wild-type embryos (Tepass *et al.* 1996; Kiehart *et al.* 2000b; Fernández *et al.* 2007; Gorfinkiel *et al.* 2009).

The amnioserosa fell apart in 14 of the 2L Dfs (Table 1). *Df(2L)69* provides an example of the effects of adhesion loss in the amnioserosa during dorsal closure (Figure 3C-C’’’). In early closure, three of the seven homozygous *Df(2L)69* embryos imaged showed disorganized and uneven cadherin distribution in the amnioserosa. By mid-closure, the amnioserosa in these three embryos appeared to disintegrate, resulting in loss of adhesion to the DME cells and distortion of canthi. The other four embryos appeared normal until mid to late closure, when the amnioserosa began to fall apart. Remarkably, all but one embryo completed closure and did so at a normal rate. Thus, *Df(2L)69* demonstrates the resilience of dorsal closure, where the morphogenic process can complete in the absence of an intact amnioserosa. No known dorsal closure gene is removed by *Df(2L)69*, thus one or more novel dorsal closure genes will be uncovered by this Df.

The remaining 13 2L Dfs that caused the amnioserosa tissue to fall apart display a range of phenotypes. Six of the 13 Dfs started dorsal closure with abnormal amnioserosa cell shapes. Another five of the Dfs had wild-type morphologies at the start of closure. The remaining two Dfs fell apart at the start of dorsal closure and it was difficult to determine if cell shapes were normal prior to tissue failure. Three of the 13 Dfs in addition to *Df(2L)69* (selected as representative, above) had a severe phenotype where the amnioserosa fell apart completely. One of these four severe Dfs (*Df(2L)54*) appeared to develop holes within the amnioserosa cells rather than at the junctions between amnioserosa cells. The remaining nine Dfs formed holes throughout the amnioserosa or in concentrated locations, which have moderate to mild effects on the completion of closure. Embryos homozygous for three of the Dfs appeared to develop holes due to increased ingressions rather than breakdown of the adherens junctions *per* *se*. We conclude that amnioserosa holes can arise via multiple mechanisms.

#### Abnormal amnioserosa cell ingressions:

During dorsal closure, the amnioserosa must maintain adhesion with neighboring amnioserosa cells even when some cells ingress into the interior of the embryo. When ingressions occur too frequently, holes can develop in the amnioserosa tissue (Mortensen *et al.* 2018). Conversely, when the ingression rate is reduced or absent, dorsal closure can slow (Toyama *et al.* 2008; Sokolow *et al.* 2012). Thus, precise regulation of ingression rate plays a key role in the efficient and successful completion of dorsal closure.

Thirteen 2L Dfs when homozygous showed changes in ingression rates and provide insight into the effect this has on dorsal closure (Table 1). *Df(2L)100* provides an example of increased ingression which lead to minor holes forming at the end of dorsal closure (Figure 3D-D’’’). Interestingly, these ingressions occurred primarily in the central amnioserosa toward the anterior end in comparison to control embryos where only 10% of ingressions occur in the central amnioserosa (Sokolow *et al.* 2012). Embryos homozygous for *Df(2L)100* also showed a delay in anterior canthus formation. The increase in ingression events at the anterior end of the dorsal opening in embryos homozygous for *Df(2L)100* may compensate for the observed lag in zipping and allowed dorsal closure to complete at a rate similar to control embryos.

As with *Df(2L)100*, 10 of the remaining 12 Dfs with ingression defects had an increase in ingression within the central amnioserosa that seemed to cause other defects such as irregular amnioserosa cell shapes, exacerbated asymmetric zipping or a wavy or jagged dorsal opening shape. Only two 2L Dfs appeared to have decreased ingressions, which lead to irregular amnioserosa cell shapes, a cigar shaped dorsal opening and slowed closure.

### Lateral epidermis phenotypes (18 Dfs)

At the onset of dorsal closure, the DME cells begin to elongate circumferentially along the dorsal-ventral axis, as the supracellular purse-strings develop and the leading edge is transformed into a smooth arc (Young *et al.* 1993; Kiehart *et al.* 2000b; Fernández *et al.* 2007). As dorsal closure initiates, successive rows of more ventral, lateral epidermal cells also elongate along the dorsal-ventral, circumferential axis. The lateral epidermis is well-organized into distinct rows of cells. The lengthening of the DME cells toward the dorsal midline and the zipping together of the two flanking lateral epidermal sheets results in a seamed, then a seamless, epithelium (Jacinto *et al.* 2002a; Das Gupta and Narasimha 2019). The bulk lateral epidermis flanking the amnioserosa resists the dorsal-ward movement and provides an opposing force (Hutson *et al.* 2003).

**Figure 4:**
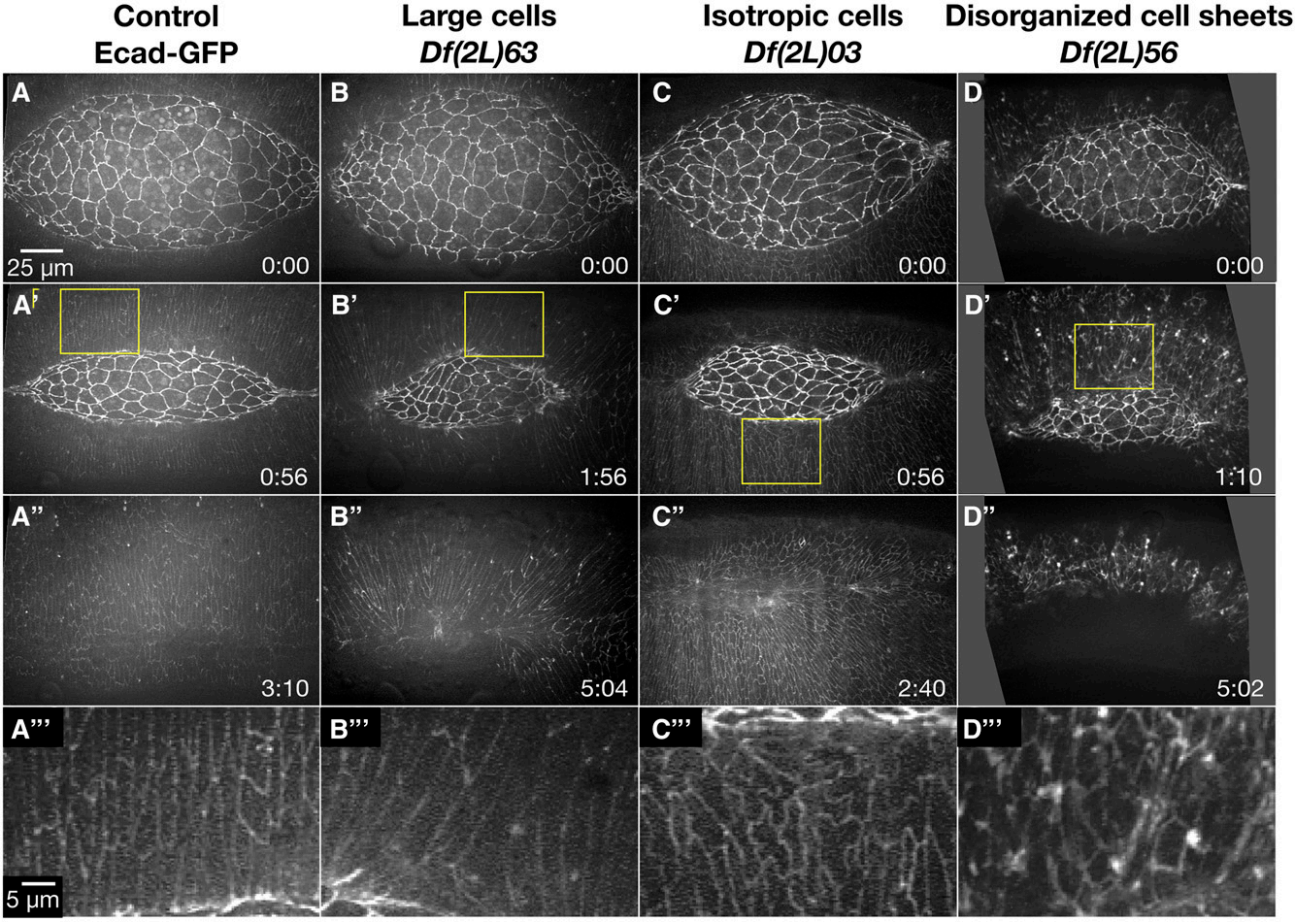
Lateral epidermal phenotypes observed in homozygous 2L Df embryos. Stills from a time-lapse sequence of Ecad-GFP labeled control embryos (A-A’’’) and homozygous Df embryos with lateral epidermal phenotypes. Homozygous *Df(2L)*63 embryos have large lateral epidermal cells (B-B’’’). Homozygous *Df(2L)03* embryos have isotropic lateral epidermal cells (C-C’’’). Homozygous *Df(2L)56* embryos have disorganized lateral epidermal cell sheets (D-D’’’). The yellow boxed areas in A’, B’, C’, and D’ are magnified in A’’’, B’’’, C’’’, and D’’’. Anterior is to the left, posterior to right. Time is in hr:min, time 0:00 is at the start of the experimental run when the height of the dorsal opening was between 75-100 µm. The scale bar in A applies to panels A-D”. The scale bar in A’’’ applies to panels A’’’-D’’’. Stills are from Suppl Movies 6-9, Supp Movies 10 and 24 provide additional examples of *Df(2L)56* and *Df(2L)63*, respectively.

Eighteen Dfs in the 2L kit had a lateral epidermal dorsal closure phenotype (Table 2). The phenotypes fell into three groups: large cells, isotropic/non-elongated cells, and disorganized lateral epidermal cell sheets (Figure 4).

**Table 2:** Deficiencies that cause lateral epidermis phenotypes (18 total)

Lateral Epidermis Phenotype	Number of Dfs	Screen Name Df(2L)n
Large cells	**5**	23, 24, 27, 63, 64
Isotropic/non-stretched cells	**11**	03, 23, 24, 25, 27, 46, 47, 54, 56, 72, 97
Disorganized cell sheets	**10**	10, 22, 25, 54, 56, 63, 64, 94, 96^A^, 99^A^

The table lists the three classes of lateral epidermal phenotypes which were observed in embryos homozygous for 2L Dfs, the number of Dfs that caused each of these phenotypes and the Dfs that caused them. Note that Dfs are denoted by chromosomal position (see Material and Methods). The corresponding Bloomington stock numbers and the FlyBase nomenclature can be found in Appendix A. Some Dfs cause phenotypes in more than one category. The superscript A for *Df(2L)96^A^* and *99^A^* indicate that these Dfs were added to the study to provide further coverage of the genomic region removed by *Df(2L)96* and *99* which could not be imaged at the time of dorsal closure due to tissue failure prior to dorsal closure.

#### Large lateral epidermal cells:

Large lateral epidermal cells are often the result of decreased cell numbers in the lateral epidermis due to either defects in cell division, or early patterning defects that cause large scale cell death due to altered cell identity in the lateral epidermis (Hughes and Krause 2001; Jankovics *et al.* 2011; Mortensen *et al.* 2018). In our 2R Df screen we also found that embryos homozygous for Dfs that cause large lateral epidermal cells had post closure scarring, likely due to altered cell identity and therefore poor segment matching during zipping. We assess size based on the cell area at the level of cell junctions, we surmise that volume is likely changing as well, but cannot rule out a redistribution of volume with the flattening of cells as volume cannot be accurately measured with the Ecad-GFP fluorescent marker.

Five 2L Dfs that had large lateral epidermal cells uncovered genes involved in mitosis or in early embryo patterning (Table 2). *Df(2L)63* provides an example of large lateral epidermal cells that elongate circumferentially along the dorsal-ventral axis (Figure 4B-B’’’). Additionally, the arrangement of these lateral epidermal cells was disorganized – the lateral epidermis did not consist of orderly arrays of distinct rows of cells. Some DME cells bunched together at the leading edge causing the cells to become teardrop-shaped. The canthi were poorly formed, and the dorsal opening became aberrantly shaped as closure proceeded to completion which resulted in scarring at the seam post-closure. Unsurprisingly, closure was delayed in comparison to controls. It is not clear whether all of these phenotypes were a consequence of the large lateral epidermal cell areas or distinct phenotypes independent of large cells. Embryos homozygous for *Df(2L)64* fully phenocopied the dorsal closure phenotype of *Df(2L)63*; therefore, the gene or genes responsible for the phenotype is likely in an interval removed by both Dfs. Indeed, we have identified *pimples* as the gene responsible for large lateral epidermal cells in *Df(2L)63* and *64* (discussed in greater detail below). In the 2R Df screen, large lateral epidermal cell shapes do correlate with scarring post-closure; however, the disorganized lateral epidermis and irregular dorsal opening shape is not commonly observed in embryos homozygous for these 2R Dfs (Mortensen *et al.* 2018). We surmise that *pimples* may work in additional pathways independent of its role in large lateral epidermal cells that are important for different aspects of the more complex dorsal closure phenotype (Stratmann and Lehner 1996).

The three remaining 2L Dfs that caused embryos homozygous for the Df to have large lateral epidermal cells (*Df(2L)23*, *24*, and *27*) all removed an early patterning gene (discussed in detail below).

#### Isotropic, non-elongated lateral epidermal cells:

At the conclusion of germband retraction in wild-type embryos, the microtubules in the lateral epidermis are reoriented along the circumferential dorsal-ventral axis and the lateral epidermal cells elongate in this direction (Young *et al.* 1993; Kaltschmidt *et al.* 2002; Jankovics and Brunner 2006).

In embryos homozygous for 11 2L Dfs the majority of the lateral epidermal cells did not elongate and remained isotropic throughout dorsal closure (Table 2, Figure 4C-C’’’). The lateral epidermal cells of *Df(2L)03* remained isotropic with “wiggly” cell perimeters in the middle (thoracic and abdominal 1-5 segments) of the embryo and segmentation of the lateral epidermis appeared to be lost in this region. However, the lateral epidermal cells at both the anterior and posterior ends of the dorsal opening elongated normally with straight cell boundaries. As closure progressed, the posterior canthus formed poorly, appearing rounded and causing the dorsal opening to become asymmetric. All embryos completed closure at rates comparable to control embryos but had some scarring. The isotropic cell shapes and lack of segmental boundaries in the middle of the embryo suggest that segment identity may be aberrant in homozygous *Df(2L)03* embryos. We speculate that aberrant cell elongation and scarring may be due to loss of segment polarity and thus poor cell matching at the dorsal midline. The “wiggly” cell perimeters observed in *Df(2L)03* is likely an additional phenotype to the isotropic lateral epidermal cell shapes as no other 2L Df, with isotropic or elongated lateral epidermal cell shapes, shared this phenotype.

Similar to *Df(2L)03*, five of the remaining ten Dfs caused homozygous embryos to include regions of isotropic (*i.e.*, non-elongated) cells in the lateral epidermis. Such regions were observed in the middle of the embryo, in the posterior half or in segmental boundaries – the remainder of the tissue had lateral epidermal cells that were properly elongated. Conversely, the lateral epidermal cells of the other five Dfs were uniformly isotropic. The hierarchy of the patterning gene in the anterior-posterior patterning pathway may explain the extent to which and the regional distribution of cells that are isotropic in shape. For example, one of the earliest patterning genes, *even-skipped* – a primary pair-rule gene, causes uniformly isotropic lateral epidermal cells, while *paired*, a secondary pair-rule gene, causes isotropic cells in the posterior half of the embryo (discussed in detail below, Schroeder *et al.* 2011; Mortensen *et al.* 2018). Only one Df with isotropic cell shapes, Df(2L)56, does not remove a gene known to be involved in segment polarity patterning or a known dorsal closure gene. This Df has the potential to uncover a novel dorsal closure gene important for the mechanics of elongation rather than cell identity, providing a new link to the pathways already known to regulate cell elongation such as *c-Jun N-terminal kinase* (*JNK*) and *Decapentaplegic* (*Dpp*, McEwen *et al.* 2000; Fernández *et al.* 2007; Lada *et al.* 2012).

#### Disorganized lateral epidermal cells:

In native dorsal closure, the lateral epidermal sheets are highly organized and have regularly spaced cell rows (reviewed in Kiehart *et al.* 2017).

**Figure 5:**
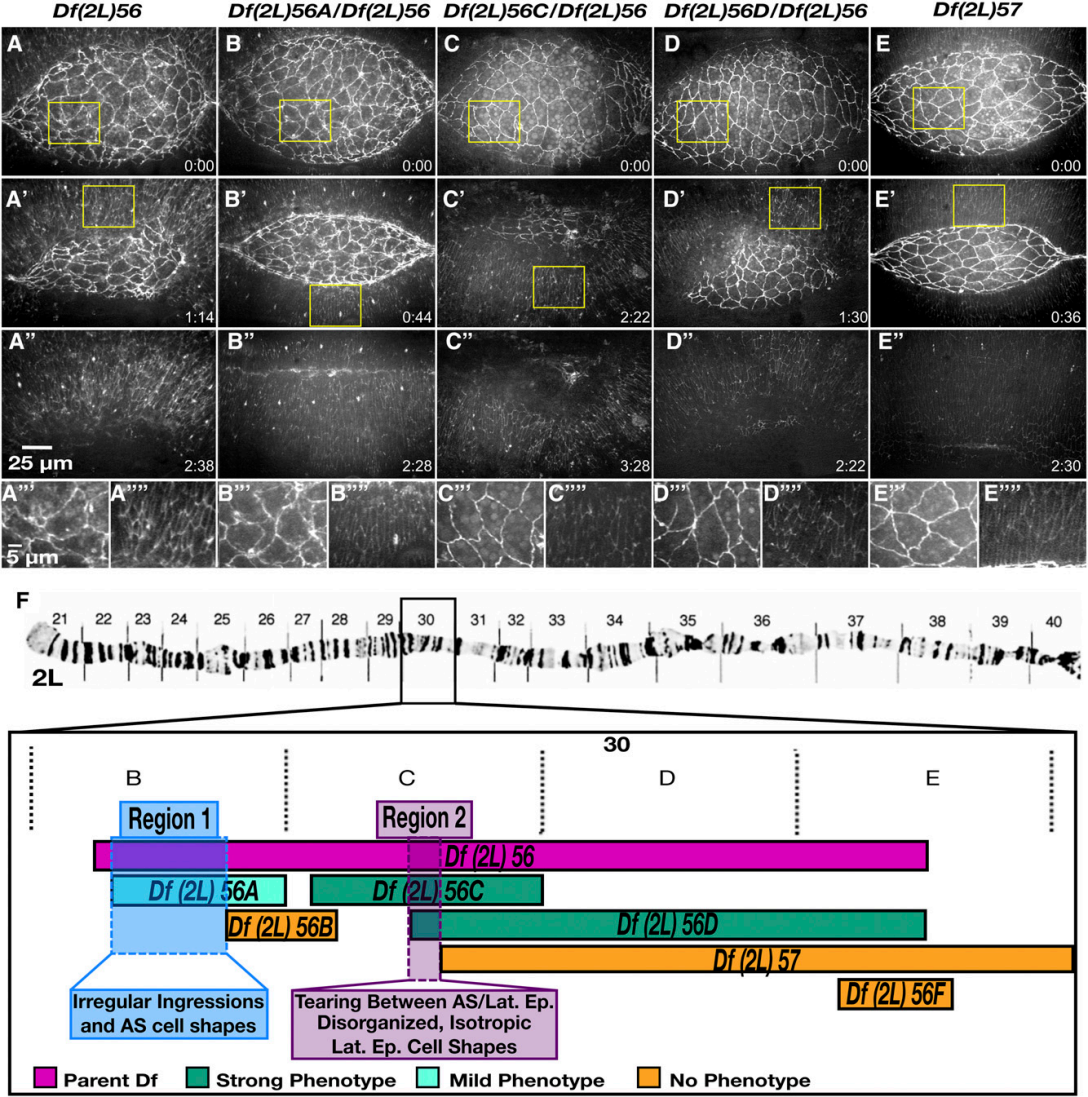
The severe and complex phenotype in embryos homozygous for Df(2L)56 can be divided into three separate phenotypes due to three distinct genomic regions. Stills from time-lapse sequence of homozygous *Df(2L)56* (A-A’’’’), transheterozygous *Df(2L)56A* / *Df(2L)56* (B-B’’’’), transheterozygous *Df(2L)56C* / *Df(2L)56* (C-C’’’’), transheterozygous *Df(2L)56D* / *Df(2L)56* (D-D’’’’) and homozygous *Df(2L)57* embryos (E-E’’’’) in a *D*E-cadherin-GFP imaging background. Homozygous embryos of *Df(2L)56* have a strong and fails dorsal closure phenotype. Transheterozygous embryos of *Df(2L)56* with *Df(2L)56A* have a mid-severity dorsal closure phenotype with irregular amnioserosa cell shapes. Embryos transheterozygous for *Df(2L)56C* / *Df(2L)56* as well as embryos transheterozygous for *Df(2L)56D* / *Df(2L)56* have a ‘strong and fails’ phenotype –the amnioserosa tears away from the lateral epidermis and the lateral epidermal cell sheet has disorganized, isotropic cell shapes. Homozygous embryos of *Df(2L)57* have no dorsal closure phenotype. The yellow boxed areas in A, A’, B, B’, C, C’, D, D’, E and E’ are magnified in A’’’, A’’’’, B’’’, B’’’’, C’’’, C’’’’, D’’’, D’’’’, E’’’ and E’’’’, respectively. A cytological map schematic of the left arm of chromosome 2 depicts the region removed in *Df(2L)56* and overlapping sub-Dfs (F). The polytene chromosome micrograph was previously published in Halsell and Kiehart (1998) and Lefevre (1976). Embryos transheterozygous for *Df(2L)56F* / *Df(2L)56* have no dorsal closure phenotypes (denoted in orange). Anterior is to the left, posterior to right. Time is in hr:min, time 0:00 is at the start of the experimental run when the height of the dorsal opening was between 75-100 µm. The scale bar in A applies to panels A-D’’ and the scale bar in A’’’ applies to A’’’-E’’’’. Stills are from Suppl Movies 10-14, Supp Mov 9 provides an additional example of *Df(2L)56*.

The organization of the lateral epidermal cell sheets was disrupted in ten of the 2L Dfs (Table 2). The degree of disorganization was highly variable: in some Dfs only the DME cells were disrupted while in others such as *Df(2L)56* the entire lateral epidermis was highly disorganized (Figure 4D-D’’’). Of the nine homozygous *Df(2L)56* embryos imaged, eight displayed very disorganized, isotropic lateral epidermal cells throughout dorsal closure. Seven of these eight homozygous *Df(2L)56* embryos also had abnormal amnioserosa cell shapes and ingressions. In late dorsal closure the amnioserosa tore away from the lateral epidermis in seven embryos and dorsal closure failed. Embryos transheterozygous for *Df(2L)56* and overlapping sub-Dfs were imaged to further characterize the genetic basis of the complex phenotype caused by *Df(2L)56*. Two distinct genomic regions were each responsible for discrete aspects of this phenotype (Figure 5). Region 1 caused irregular amnioserosa cell borders with abnormal ingression. The region responsible for the irregular amnioserosa cell shapes was narrowed down to five genes and it is possible that further analysis will establish whether the same gene(s) within that interval cause both the irregular cell shape and the ingression defects observed or are due to different genes in the interval. Region 2 caused disorganized, isotropic lateral epidermal cell shapes as well as the tearing between the amnioserosa and lateral epidermis resulting in dorsal closure failure. This genomic region included four genes shared by sub-Dfs *Df(2L)56C* and *Df(2L)56D* (Figure 5 C-E), but not shared by *Df(2L)57*. Region 2 included the 18 genes only shared by *Df(2L)56* and *Df(2L)56D* (Figure 5 D-E). Therefore, the irregular amnioserosa cell shapes and ingressions phenotype of embryos homozygous for *Df(2L)56* are due to the deletion of a different set of genes than those that cause the tearing of the amnioserosa from the lateral epidermis and the disorganized, isotropic lateral epidermal cell shapes. However, the lateral epidermal phenotype has not been genetically separated from the tearing phenotype. Further investigation into the four genes in Region 2 will determine whether the lateral epidermis and tearing phenotypes are due to the same gene(s) or are two separate phenotypes, independent of one another.

### Zipping and canthus phenotypes (33 Dfs)

The onset of closure is marked by the concerted, dorsal-ward movement of the DME cells and formation of the canthi – the previously oblong dorsal opening is transformed into an eye-shape as seams form at the opening’s anterior and posterior ends and zipping ensues. As zipping progresses the DME cells from opposing sheets are joined at the dorsal midline to form a seamed, then seamless, epithelium (Jacinto *et al.* 2000; Das Gupta and Narasimha 2019). Each DME cell is transcriptionally distinct from its neighbor due to positional inputs from signaling pathways, such as JNK and the transcriptional regulatory networks for anterior-posterior patterning (Perrimon and Desplan 1994; Mallo and Alonso 2013; Rousset *et al.* 2017) . For a seamless epithelium to form, the DME cell must be matched with the transcriptionally identical DME cell on the opposing lateral epidermal sheet. Filopodia and lamellipodia at the leading edge of these cells aid in cell matching by reaching across the divide as the DME cells are drawn into the canthus. Upon identifying their match, the lamellae and filopodia form sites of adhesion that ensure the cells are appropriately paired at the seam (Jacinto *et al.* 2000; Eltsov *et al.* 2015). Loss of these cellular protrusions can cause mismatching of segments that results in scarring at the seam (Jacinto *et al.* 2000; Gates *et al.* 2007). During normal closure, the width to height ratio of the dorsal opening slowly and monotonically increases as zipping progresses from both sides of the embryo. Thus changes in the overall shape of the dorsal opening (*i.e.*, ratio of width to height) and/or changes in canthus morphology are indicative of alterations in zipping (Hutson *et al.* 2003; Jankovics and Brunner 2006; Peralta *et al.* 2007).

**Figure 6:**
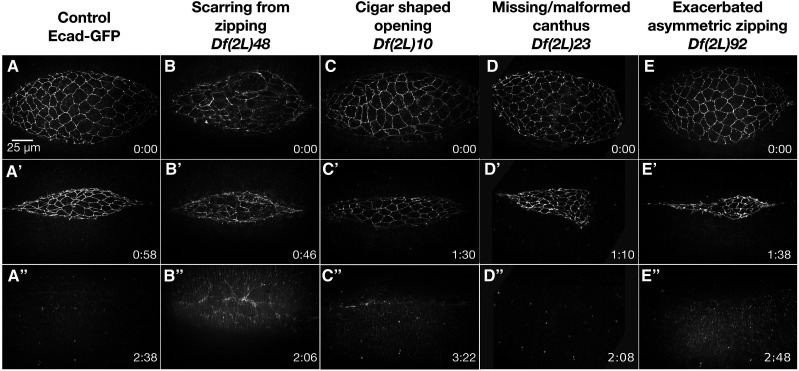
Zipping and canthus phenotypes observed in homozygous 2L Df embryos. Stills from time-lapse sequences of Ecad-GFP labeled control embryos (A-A”) and homozygous Df embryos with zipping and or canthus defects. *Df(2L)48* embryos show scarring from zipping (B-B’’). *Df(2L)10* embryos have a cigar-shaped dorsal opening (C-C’’). *Df(2L)23* embryos are missing the posterior canthus (D-D’’). *Df(2L)92* embryos have exacerbated asymmetric zipping: the anterior canthus zips faster than the posterior (E-E’’). Note that the timepoint was chosen as it illustrates the extreme asymmetry which is driven by increased ingression; however, a smaller more apical z-projection would show seam formation, but doesn’t illustrate zipping as well. Anterior is to the left, posterior to the right. Time is in hr:min, time 0:00 is at the start of the experimental run when the height of the dorsal opening was between 75-100 µm. The scale bar in A applies to all micrographs. Stills are from Suppl Movies 15-19.

Thirty-three 2L Dfs had zipping and/or canthus defects that manifested as scarring post-closure, cigar-shaped dorsal openings, missing or malformed canthi, and exacerbated asymmetry in zipping (Table 3, Figure 6).

**Table 3:** Deficiencies with zipping/canthus phenotypes (33 total)

Zipping/Canthus Phenotype	Number of Dfs	Screen Name Df(2L)n
Scarring from zipping	**14**	10, 17, 25, 27, 28, 29, 43, 48, 51, 55, 63, 64, 68, 99^A^
Cigar shaped opening	**10**	01, 09, 10, 12, 19, 24, 25, 38, 51, 97
Missing/malformed canthus	**14**	03, 22, 23, 25, 27, 37, 54, 63, 64, 72, 84, 94, 97, 100
Exacerbated asymmetric zipping	**4**	08, 17, 52, 92

Listed are the four classes of zipping and canthus phenotypes which were observed in embryos homozygous for 2L Dfs, the number of Dfs that caused the phenotypes and the Dfs that caused them. Dfs are denoted by chromosomal position (see Material and Methods) – the corresponding Bloomington stock numbers and the FlyBase nomenclature can be found in Appendix A. Some Dfs cause phenotypes in more than one category. The superscript A for *Df(2L)99^A^* indicates that this Df was added to the study to provide further coverage of the genomic region removed by *Df(2L)99* which could not be imaged at the time of dorsal closure due to tissue failure earlier in development.

#### Scarring from zipping:

Scarring is an aberration in the formation of the continuous dorsal epidermis that characterizes wild-type closure. In control embryos the DME cells constrict at their dorsal borders as they are zipped into the canthus. This constriction quickly relaxes and the epithelial cells have near uniform widths of approximately 3.4 ± 0.7 µm as the canthi continue to proceed along the anterior posterior axis, toward the middle of the amnioserosa (Kiehart *et al.* 2000b; Das Gupta and Narasimha 2019). When scarring occurs some of the DME cells remain constricted at their dorsal borders. This causes bunching or pinching along the seam while the remaining cells appear to over-relax – they are stretched and become elongated along the anterior-posterior axis.

Embryos homozygous for each of fourteen 2L Dfs formed scars during zipping and never formed a seamless epithelium for the duration of imaging, approximately thirty minutes to one hour after closure had completed (Table 3). Five of the fourteen Dfs had irregular lateral epidermal cell shapes and/or organization which may have contributed to the scarring post-closure. The other eight Dfs progressed through closure with properly shaped and organized lateral epithelia but developed scarring post-closure as exemplified by images of homozygous *Df(2L)48* embryos (Figure 6B-B’’). These defects were likely due to problems with adhesion between the DME cells from contralateral sides of the embryo as they zipped together and/or the transition from a seamed to a seamless epithelium. In addition to scarring, embryos homozygous for *Df(2L)48* also had irregularly-shaped amnioserosa cells – the cadherin junctions broke down in five of the six embryos imaged, forming holes in the amnioserosa tissue. Remarkably, only one embryo did not complete closure, the other five embryos completed closure at near native rates. Adhesion appeared to be disrupted in the amnioserosa tissue and may also have affected DME cell adhesion between the contralateral leading edges during zipping, which may have led to scarring. Further investigation into the mechanism of the scarring observed in *Df(2L)48* may provide insight into proper cell matching and adhesion.

#### Cigar-shaped dorsal opening:

As closure progresses the change in height and width of the dorsal opening are closely coordinated such that as zipping occurs from both ends of the dorsal opening, the curvature of the purse-strings remains nearly constant until the final stages of closure. Slowed zipping can often result in a cigar-shaped dorsal opening (Hutson *et al.* 2003; Jankovics and Brunner 2006; Wells *et al.* 2014).

Ten 2L Dfs had slowed zipping that resulted in a cigar-shaped opening (Table 3). Embryos homozygous for *Df(2L)10* had a severe cigar-shaped dorsal opening throughout closure (Figure 6C-C’’). The DME cells bunched together sporadically along the leading edge, while cells in-between became elongated along the anterior-posterior axis. This disorganization of the DME may have impaired zipping – when entering the canthus these bunched and stretched cells could not properly pair and closure was slowed. Not surprisingly, scarring was observed post-closure in all seven *Df(2L)10* embryos imaged.

The majority of embryos homozygous for the remaining nine Dfs that caused a cigar shaped dorsal opening were similar to *Df(2L)10* – the cigar shape developed in early- to mid-closure. Just two Dfs developed a cigar shape only late in closure. All 2L Dfs that caused a cigar shape had other dorsal closure defects – irregular amnioserosa cell shapes or problems with canthus formation and morphology – that likely contributed to the development of the cigar shape. While the cigar shaped dorsal opening of these ten Dfs appeared to be the consequence of upstream defects, the Dfs are instructive to understand how either tissue, the lateral epidermis or amnioserosa, influence zipping.

#### Missing or malformed canthus:

The morphology of the canthus can be instructive about the efficiency of zipping, as well as about adhesion between cells of the lateral epidermis (Peralta *et al.* 2007; Peralta *et al.* 2008; Lu *et al.* 2015).

Embryos homozygous for 14 Dfs in the 2L Df kit displayed a range of defects in canthus formation and morphology (Table 3). Embryos homozygous for nine of these 14 Dfs did not form canthi normally – canthus formation was delayed in six Dfs and one canthus never formed in three Dfs. Embryos homozygous for three of the Dfs that were delayed in forming a canthus also had abnormalities in canthus morphology, with canthi that appeared rounded. The canthus morphology was also rounded in embryos homozygous for five of the 14 Dfs that did form canthi at the onset of closure. In some cases, only one canthus was affected. In embryos homozygous for three Dfs only the anterior canthus had defects, which may or may not have been secondary to problems with head involution, whereas in embryos homozygous for three Dfs only the posterior canthus displayed defects. For example, the posterior canthus did not form in two of the embryos homozygous for *Df(2L)23* (Figure 6D-D’’), in another four embryos the posterior canthus formation was delayed and remained blunt throughout closure. Regardless, closure completed at nearly native rates in all six embryos imaged. Homozygous *Df(2L)23* embryos also had defects in the shape of lateral epidermal cells – they remained isotropic throughout the duration of closure and failed to elongate along the circumferential, dorsal-ventral axis. Previous studies show that canthus formation at the anterior and posterior ends of the dorsal opening occurs via different mechanisms as the morphology at each end following germband retraction is unique and each canthus must form over a different underlying tissue (Wells 2013). Prior to canthus formation, at the end of germband retraction, the anterior margin of the dorsal opening is very broad and blunt and the canthus forms over the yolk. In contrast, the posterior end of the dorsal opening is more rounded, there is little to no distance between the flanking lateral epidermal sheets and the posterior canthus forms over the gut. Further investigation into the 2L Dfs with canthus defects, especially those that affect only one canthus, will provide valuable insight into the different mechanisms important for canthus formation at each end of the dorsal opening.

#### Exacerbated asymmetry in zipping:

In wild-type embryos, zipping during dorsal closure is asymmetric with the anterior canthus zipping at a rate 45% faster than the posterior canthus. The rate of zipping can be increased at either canthus by experimentally inhibiting zipping at the other canthus through laser microsurgery (Peralta *et al.* 2007).

Embryos homozygous for four 2L Df stocks showed exacerbated asymmetry in zipping (Table 3) – in each case, both canthi were well formed, but one zipped much faster than the other. The exacerbated asymmetry in zipping of these four 2L Dfs was due to impaired or reduced zipping at the opposite canthus. Interestingly, this phenotype was not cataloged in our previous 2R Df screen and the phenotype is rare in comparison to the other dorsal closure defect categories, only occurring in four 2L Dfs. Additionally, this phenotype appeared to be linked to irregular ingressions – embryos homozygous for all four 2L Dfs with exacerbated asymmetric zipping had an increase in ingression in the anterior or posterior half of the dorsal opening, that resulted in faster zipping of the canthus adjacent to the increased ingressions. For example, *Df(2L)92* homozygous embryos zipped primarily from the anterior canthus in concert with increased ingression events in the anterior half of the amnioserosa (Figure 6E-E’’). The anterior canthus of embryos homozygous for *Df(2L)52* also zipped faster than the posterior. The other two Dfs that caused exacerbated asymmetric zipping primarily zipped from the posterior canthus. Therefore, this phenotype is not unique to the anterior or posterior canthus but is consistent with the influence of locally increased ingression on the rate of zipping. What causes local increases in ingression remains a mystery – further insight into this will require identifying which gene or genes are responsible for the Df phenotypes.

### Phenotypes at the interface between the amnioserosa and lateral epidermis (18 Dfs)

The adhesion between the PAS and DME cells is very important for dorsal closure. Following germband retraction the leading edge of the DME cells is scalloped or wavy. As the supracellular actin and myosin purse-strings form, scalloping disappears and the leading edges form a smooth arc (Kiehart *et al.* 2000b). During this transition, the morphology of the PAS and DME cells shifts so that the PAS cells tuck under the neighboring epithelium and the two cells become reciprocally wedge-shaped. This change in shape maximizes the surface area of contacts between the PAS and DME cells. Integrins accumulate and stabilize this junction (Narasimha and Brown 2004; Wada *et al.* 2007). In the absence of integrin, this change in morphology does not occur and adhesion is lost, causing the amnioserosa to tear away from the lateral epidermis.

**Figure 7:**
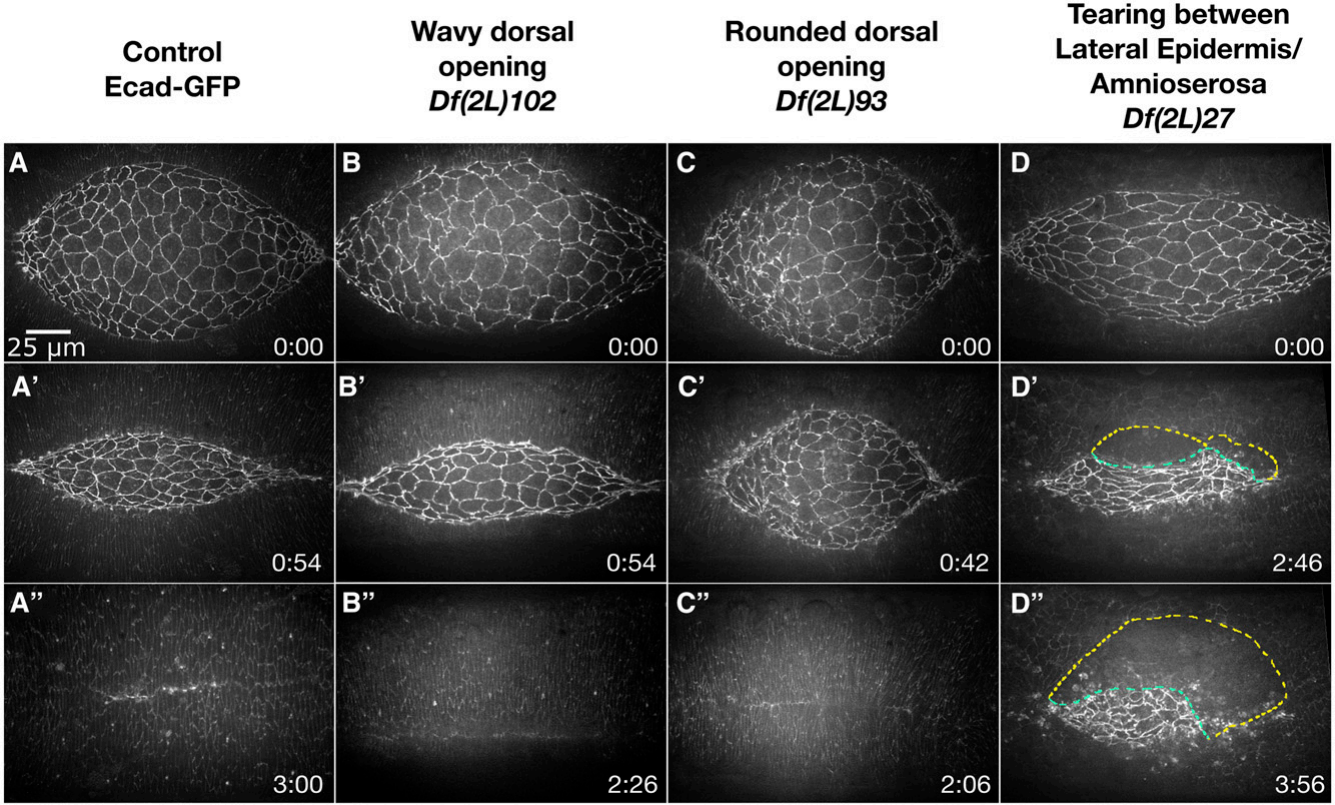
Phenotypes at the interface of the peripheral amnioserosa and dorsal-most epithelium observed in homozygous 2L Df embryos. Stills from a time-lapse sequence of Ecad-GFP labeled control embryos (A-A”) and homozygous Df embryos with phenotypes at the interface of the peripheral amnioserosa and dorsal-most epithelium. A wavy dorsal opening is observed in *Df(2L)102* embryos (B-B’’). *Df(2L)93* embryos have a rounded dorsal opening (C-C’’). The peripheral amnioserosa tears away from the dorsal-most epithelium in *Df(2L)27* embryos (D-D’’). The yellow dashed line mark the border of the lateral epidermis and the green dashed line marks the amnioserosa edge. Anterior is to the left, posterior to right. Time is in hr:min, time 0:00 is at the start of the experimental run when the height of the dorsal opening was between 75-100 µm. The scale bar in A applies to all micrographs. Stills are from Suppl Movies 20-23, note that Supp Mov 30 provides an additional example of *Df(2L)27*.

Embryos homozygous for eighteen 2L Dfs had phenotypes indicative of defects at the interface between the amnioserosa and lateral epidermis (Table 4). These Dfs caused jagged or wavy dorsal openings, rounded dorsal openings, and/or tearing along the interface between the amnioserosa and lateral epidermis border (Figure 7).

**Table 4:** Deficiencies that cause phenotypes at the interface between the amnioserosa and lateral epidermis (18 total)

Phenotype at Interface Between amnioserosa and lateral epidermis	Number of Dfs	Screen Name Df(2L)n
Jagged or wavy dorsal opening	**8**	08, 28, 29, 42, 43, 92, 93, 102
Rounded dorsal opening	**3**	45, 72, 93
Tearing along the amnioserosa/lateral epidermis border	**8**	27, 35, 37, 46, 47, 56, 61, 99^A^

Listed are the three distinct phenotypes that affect the interface between the amnioserosa and the lateral epidermis, the number of Dfs that caused the phenotypes and the Dfs that caused them. The Dfs are denoted by chromosomal position (see Material and Methods), the corresponding Bloomington stock numbers and the FlyBase nomenclature can be found in Appendix A. Some Dfs cause phenotypes in more than one category. The superscript A for *Df(2L)99^A^* indicates that this Df was added to the study to provide further coverage of the genomic region removed by *Df(2L)99* which could not be imaged at the time of dorsal closure due to tissue failure prior to dorsal closure.

#### Jagged or wavy dorsal opening:

At the onset of dorsal closure in wild-type embryos, the scalloped or wavy leading edge resolves into a smooth arc with the accumulation of actin and myosin. A wavy dorsal opening suggests that the actin and/or myosin of the purse-strings are absent or reduced and thus scalloping at the leading edges persists. As closure progresses the wavy shape can become more severe and a jagged shape results (Kiehart *et al.* 2000; Laplante and Nilson 2011).

In embryos homozygous for eight 2L Df stocks, the leading edge of the lateral epidermis remained wavy and failed to resolve into a smooth arc; of these, four developed into a more severe jagged shape (Table 4). For this reason, we have updated the wavy dorsal opening category from the 2R Df screen to include “jagged”. Embryos homozygous for *Df(2L)102* are a representative example of the four Dfs that had a wavy dorsal opening throughout dorsal closure that did not become severely jagged and closure rates were similar to controls (Figure 7B-B’’).

The development of the more severe jagged dorsal opening shape in embryos homozygous for the remaining four Dfs may indicate a change in force dynamics between the amnioserosa and lateral epidermis, changes in the material properties of the amnioserosa and/or lateral epidermis, changes in the structure or function of the purse-strings or some combination of all four. Embryos homozygous for *Dfs(2L)28* and *29* removed the known dorsal closure gene *echinoid*, a cell adhesion molecule required for proper assembly of the purse-strings. The initially wavy, then jagged dorsal opening shape of these Dfs was consistent with the published dorsal closure phenotype reported for loss of *echinoid* (Laplante and Nilson 2011). The other two Dfs that had a jagged dorsal opening shape, *Dfs(2L)42* and *43*, both removed the known dorsal closure genes *mummy** (mmy)* and *Sec61 α subunit* (*Sec61α*, Schimmelpfeng *et al.* 2006; Wang and Ward 2010). It is important to note that the published dorsal closure phenotypes of *mmy* and *Sec61α* are independently more severe than that of the Dfs removing both genes. While the dorsal closure phenotype of *Df(2L)43* was slightly more severe, with irregular amnioserosa cell shapes and scarring post closure, the development and morphology of the jagged dorsal opening shape was indistinguishable between *Dfs(2L)42* and *43*. Thus, either the loss of *mmy* in conjunction with *Sec61α* or an additional gene removed by both Dfs are likely the cause of the jagged dorsal opening shape. This inconsistency is discussed in greater detail below when discussing the Dfs removing known dorsal closure genes.

#### Rounded dorsal opening:

A rounded dorsal opening has a height to width ratio closer to one (∼0.7 *vs.* control value of ∼0.3, measured from purse-string to purse-string along the circumferential dorsal-ventral axis and from canthus to canthus along the anterior-posterior axis). While the dorsal opening is rounded, canthi do form at the anterior and posterior ends of the opening where zipping occurs. A rounded dorsal opening is likely due to increased zipping, decreased amnioserosa contractions and/or ingressions, or some combination of the two.

Three 2L Df stocks caused a rounded dorsal opening shape (Table 4). Embryos homozygous for *Df(2L)93* had a rounded dorsal opening and abnormal amnioserosa cell shapes in early to mid-dorsal closure and the rate of closure appeared slow (Figure 7C-C’’). In mid- to late-stages of closure, the amnioserosa developed holes, primarily in the anterior half, and the margins of the opening became wavy while closure rates increased. It seems that the rounded dorsal opening shape in the case of *Df(2L)93* was likely due to decreased amnioserosa contractions and ingressions during the first half of dorsal closure. In mid- to late-dorsal closure stages when the amnioserosa tissue breaks down, the dorsal opening transitioned from a rounded dorsal opening shape to a wavy shape and closure completed within a timespan comparable to controls. In contrast, embryos homozygous for *Df(2L)45* had a normal dorsal opening shape through mid-dorsal closure; however, in late closure the dorsal opening became very rounded. *Df(2L)45* removed the known dorsal closure gene *Rab30*, a small GTPase that is a transcriptional target of JNK signaling that is believed to play a role in exocytosis –11.5% of embryos injected with dsRNAi targeting *Rab30* show a dorsal open cuticle phenotype (Thomas *et al.* 2009). Defects in the dorsal cuticle are not directly comparable to our Df images due to the inability to observe cellular and tissue level kinematics and dynamics. Furthermore, embryos homozygous for *Df(2L)45* are zygotically null for *Rab30* whereas RNAi mediated knockdown may only partially block Rab30 function, may have off target effects or a combination of both. For these reasons, further investigation will be required to determine whether loss of *Rab30* is responsible for the observed rounded dorsal opening in late stage dorsal closure embryos homozygous for *Df(2L)45*. The third Df that caused a rounded dorsal opening, *Df(2L)72*, had isotropic lateral epidermal cell shapes. The dorsal opening became rounded early in closure and persisted throughout. The gene responsible for the aberrant lateral epidermal cell shapes of *Df(2L)72* has been identified and is discussed below.

#### Tearing along the amnioserosa and lateral epidermis border:

Adhesion at the specialized, integrin dependent junction between the DME and PAS cells must be strong to hold the two tissues together as opposing forces from the bulk of the lateral epidermal cells oppose forces generated by the contracting amnioserosa. When adhesion and cytoskeletal proteins are altered in the DME and/or PAS cells, the integrity of this junction is compromised (Young *et al.* 1993; Kaltschmidt *et al.* 2002; Franke *et al.* 2005; Jurado *et al.* 2016).

In embryos homozygous for eight 2L Dfs, there was tearing between the DME and PAS cells (Table 4). For example, in five of the seven embryos homozygous for *Df(2L)27* that were imaged, the amnioserosa tore away from the lateral epidermis and the canthi became malformed (Figure 7D-D’’). Surprisingly, three of the five embryos that had tearing go on to complete dorsal closure, but more slowly than controls. Additionally, six of the seven embryos imaged had large, isotropic (non-elongated) lateral epidermal cell shapes throughout dorsal closure. It is not clear whether the lateral epidermal cell shapes contributed to the tearing phenotype; however, some mutants that affect lateral epidermal cell shapes have been found to also disrupt the stability of the junction between the lateral epidermis and amnioserosa (McEwen *et al.* 2000; Kaltschmidt *et al.* 2002; Morel and Martinez Arias 2004). Further analysis with overlapping Dfs is required to determine whether these phenotypes are genetically distinguishable.

Four of the seven other 2L Dfs with tearing between the DME and PAS cells also had lateral epidermal defects either with isotropic cells or disorganized lateral epidermal cell sheets. Two other Dfs tore at the onset of dorsal closure, just as the DME cells were elongating. Tearing between the DME and PAS cells was one of the most severe dorsal closure defects observed and the majority of embryos with this defect failed closure.

### Deficiencies not imaged

Nine Dfs in the 2L Df kit could not be imaged as crosses to the imaging line did not generate progeny (denoted ‘Not Imaged’ in Appendix A). Either flies of the genotype *Df(2L)n* / CyO,twist-Gal4::UAS2xEGFP were inviable or the *inter-se* crosses of *Df(2L)n* / CyO,twist-Gal4::UAS2xEGFP were infertile, *i.e.*, females laid unfertilized eggs or no eggs at all. Two of these nine 2L Dfs removed the known haploinsufficient gene *dpp* (*Df(2L)13* and *15*), thus without a duplication of *dpp* the Df was not viable (Padgett *et al.* 1987; Wharton *et al.* 1996). The duplication was present in the Df kit stock but was lost during the crossing scheme required for our screen. Similarly, the *Df(2L)62* stock had a duplication for the ribosomal genes *RpL9* and *RpS27A*, without which the Df had a dominant *Minute* phenotype that lead to poor fertility and viability (Marygold *et al.* 2007). Three other Dfs (*Df(2L)18*, *30* and *31*) removed genes that were incompatible with the CyO balancer – *Df(2L)18* removed *duox*, which was responsible for the curly wing of CyO (Hurd *et al.* 2015). *Df(2L)30* and *31* overlapped by one gene, *dpy*, which has a prominent role in wing development and was incompatible with the *duox* mutation on the CyO balancer. Embryos homozygous for the final three of nine 2L Dfs, *Df(2L)81*, *85* and *86* could not be recovered in an imaging line, but the genetic reason for this was unclear. These three Dfs overlapped by a 4 gene region. However, none of these genes were haploinsufficient or *Minute* and the stocks were maintained over a CyO or SM6a balancer without the apparent addition of a duplication. This suggests that there may be interactions with the overexpression of cadherin, or GFP in general, from the twist-Gal4::UAS2xEGFP and ubi-cadherin-GFP transgenes. We crossed these Dfs to another marked balancer, CyO,Act-GFP, that does not overexpress GFP and has been found to be more widely tolerated by unhealthy stocks in our hands. However, the Dfs were still incompatible. Additionally, we tried another imaging line, sGMCA, which contains a GFP tagged actin-binding domain of Moesin, with the twist-Gal4::UAS2xEGFP marked balancer. Unfortunately, the Dfs again were incompatible. Fortunately, there was one Df stock available outside of the 2L Df kit that overlaps with *Df(2L)81* and *86* by 16 genes. This Df stock was added to the screen and is denoted as *Df(2L)86^A^*. This Df was easily crossed into the imaging line and had a very weak zipping phenotype, where three out of seven embryos imaged demonstrated a slightly cigar-shaped dorsal opening. Altogether these nine Dfs remove 76 genes not removed by other Dfs in the 2L Df kit. For the screen to be truly saturating we would need to find alternatives to test these remaining genes.

### Tissue failure prior to dorsal closure

Embryos homozygous for six Dfs from the Bloomington 2L kit were found to have major tissue failure prior to dorsal closure to the point where dorsal closure tissues were not recognizable. These six Dfs delete 324 genes on the 2L that are not removed by other Dfs in the kit. To partially compensate, we added five Dfs to the screen, which collectively deleted 79 of the 324 genes otherwise inaccessible to the study of dorsal closure because of early tissue failure. These Dfs are noted by a superscript letter in Appendix A and have the same 2L Df number as the kit Df that the new Df spans. Three of these Dfs had no dorsal closure phenotype, while two of these Dfs (*Df(2L)96^A^* and *Df(2L)99^A^*) had mid-severity to severe dorsal closure phenotypes and would have otherwise been missed by the screen (see Tables 2-4 and Appendix A).

### Deficiencies with published dorsal closure genes

Of the 108 Dfs analyzed in the screen of 2L, 38 removed a gene or genes previously published to cause a dorsal closure defect when deleted or knocked down, referred to as “known dorsal closure genes.” We were able to image dorsal closure stage embryos homozygous for 30 of these 38 Dfs. Four of these Dfs demonstrated phenotypes comparable to the published phenotype of the known dorsal closure gene. Five Dfs caused a more severe dorsal closure phenotype than the published phenotype of the known dorsal closure gene that was removed, suggesting that the Df removed an additional gene or genes important for dorsal closure. For example, *Df(2L)46* and *47* removed the known dorsal closure gene *wingless* (*wg*/*Wnt-1*), which is important for lateral epidermal cell morphology, canthus formation, and the formation of the actin and myosin purse-strings (McEwen *et al.* 2000; Kaltschmidt *et al.* 2002; Morel and Martinez Arias 2004). These Dfs also demonstrated a more severe phenotype in which the PAS and DME cells lose adhesion and dorsal closure fails. This may be due to the loss of multiple *wnt* genes as both Dfs remove *Wnt-4*, *6* and *10* in addition to *wg**/Wnt-1*.

Three Dfs caused a weaker phenotype than that caused by the dorsal closure gene or genes removed. *Df(2L)42* and *43* both remove two known dorsal closure genes, *mmy* and *Sec61α*. Loss of *mmy* or *Sec61α* causes a similar strong published dorsal closure phenotype in immunostained embryos where the gut and brain extrude through a dorsal hole (Schimmelpfeng *et al.* 2006; Wang and Ward 2010). This phenotype is also published for *Df(2L)42*; however, in our live imaging experiments of *Df(2L)42* and *43* we observed a mid-severity phenotype with a jagged dorsal opening and increased ingression. Homozygous *Df(2L)43* embryos additionally showed irregular amnioserosa cell shapes and scaring post closure and two of the seven embryos imaged did fail to complete dorsal closure due to the amnioserosa falling apart. As this appears to be an adhesion defect in the amnioserosa it is possible that the less penetrant and weaker dorsal closure phenotypes of *Df(2L)42* and *43* are due to rescue of the strong *mmy* and *Sec61α* dorsal closure defects by the overexpression of *D*E-cadherin by the imaging line used in our study. We observe instances of this in our 2R Df screen (Mortensen *et al.* 2018). The third Df, *Df(2L)78*, had a weak dorsal closure phenotype while p-element insertion alleles of the known dorsal closure gene removed, *wing blister* (*wb*), causes a cuticular dorsal hole (Martin *et al.* 1999). While the published phenotype is not directly comparable to the live imaging analysis used in this study, the weak phenotype of *Df(2L)78* would not result in a cuticular dorsal hole and therefore is classified as less severe than the dorsal closure gene removed. Again, the overexpression of *D*E-cadherin by the imaging line could rescue or reduce the dorsal closure defects caused by loss of *wb*, which encodes a laminin alpha chain and mediates cell adhesion. Alternatively, the Df may have removed one or more genes that interact with *wb*, potentially suppressing the effects of loss of zygotically expressed *wb* and resulting in the reduced phenotype. Live imaging of these three Dfs in different imaging backgrounds as well as Dfs *in trans* with null alleles of the known dorsal closure genes they remove will be required to determine why the Df has a less severe phenotype than that reported for the known dorsal closure genes removed.

Seven additional dorsal closure Dfs removed multiple known dorsal closure genes, thus the phenotype observed could not be directly compared to the published dorsal closure phenotypes. Four more dorsal closure Dfs that removed known dorsal closure genes could not be directly compared to the published phenotype for various other reasons: the previous study used the terminal cuticular dorsal hole as a read out for dorsal closure defects, the mutations analyzed used RNAi knock-down, or the phenotype was described in combination with the loss of another gene or loss of both the maternal and zygotic expression. Eight of the Dfs removing known dorsal closure genes could not be imaged in dorsal closure either due to tissue failure prior to dorsal closure (*Df(2L)41*, *95*, *96*, and *99*) or the Df was unable to be crossed into the imaging background (*Df(2L)13*, *15*, *30*, and *62*).

Finally, eight Dfs did not cause a penetrant dorsal closure phenotype even though a known dorsal closure gene is removed. This is consistent with the 2R Df screen where eight of the thirty-seven Dfs which remove known dorsal closure genes do not show a discernable dorsal closure phenotype. These dorsal closure phenotypes are described from a maternal effect experiment, by using dominant-negatives, or RNAi, or have low penetrance of the mutant phenotype. The Df is a complete loss of the zygotic gene’s expression and we only classify a Df as a dorsal closure Df if the penetrance is 50% or higher. Thus, in these cases it is not surprising that the Dfs do not phenocopy the known dorsal closure gene removed. The weaker phenotype of only one of the eight Dfs, *Df(2L)78*, cannot be explained by these criteria and is therefore listed as having a weaker phenotype than the known dorsal closure gene removed (described in detail above). More information can be found in Appendix A for each Df removing a known dorsal closure gene.

### Identifying the individual genes that cause the Df’s dorsal closure phenotype based on known phenotypes of gene families and pathways

One of the strengths of a visual screen is the ability to use known phenotypes of a gene family to aid in identifying the gene responsible for a similar phenotype. Through phenotype comparisons new genes in pathways can be identified. This was certainly true for the Heidelberg Screen where genes involved in patterning were identified by changes in cuticle structure that correlated with changes in segment identity (Nüsslein-Volhard and Wieschaus 1980). Using this approach, we have been able to immediately identify a number of new dorsal closure loci.

#### Loss of pimples causes the large lateral epidermal cells in homozygous Df(2L)63 and Df(2L)64 dorsal closure embryos:

**Figure 8:**
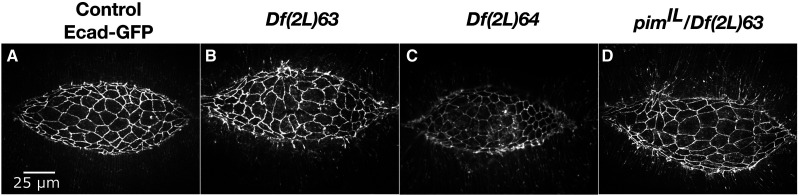
Deletion of *pim* is responsible for the large lateral epidermal cells of homozygous *Df(2L)63* and *Df(2L)64* embryos. Stills from a time-lapse sequence of mid-dorsal closure stage, Ecad-GFP labeled control (A), homozygous *Df(2L)63* (B), *Df(2L)64* (C) and transheterozygous *pim*^*IL*^ / *Df(2L)63* (D) embryos. Anterior is to the left, posterior to right. The scale bar in A applies to all micrographs. Stills are from Suppl Movies 24-27, Supp Mov 7 provides an additional example of *Df(2L)63*.

We were able to quickly identify *pimples* (*pim*) as the gene responsible for the large lateral epidermal cells in *Df(2L)63* and *64*. This phenotype was very similar to those found in embryos homozygous for *Df(2R)60* and *61*, where we identified *three-rows* (*thr*) deletion as the cause of the large lateral epidermal phenotype (Mortensen *et al.* 2018). Thr is a subunit of Separase, a trimeric complex that is important for sister chromatid separation (Philp *et al.* 1993; Pandey *et al.* 2005). Because embryos homozygous for *Df(2L)63* and *64* had a similar phenotype, we looked for genes that similarly contribute to mitosis and that were removed by both Dfs. *pim* was a clear candidate locus because it also encodes a subunit of the trimeric securin-separase complex (Stratmann and Lehner 1996; Jager *et al.* 2001). To test this notion, we created embryos transheterozygous for the *pim* null allele (*pim*^*IL*^*)* and *Df(2L)63*. These embryos also had large lateral epidermal cell shapes, DME cells bunching along the leading edge in late closure, and slight scarring post-closure (Figure 8). These phenotypes were indistinguishable from the phenotype of homozygous *Df(2L)63* and *64* embryos as well as transheterozygous *Df(2L)63* / *64* embryos. We conclude that loss of *pim* was responsible for the observed dorsal closure phenotypes in these Dfs. Additionally the large lateral epidermal cells in *pim*^*IL* / *Df(2L)63* or *64* embryos were associated with slight scarring, as previously described for *thr*^*1*^* / Df(2R)60* and *thr*^*1*^* / Df(2R)61* (Mortensen *et al.* 2018), further supporting the conclusion that a defect causing large epidermal cells leads to slight scarring post-closure. However, the *pim* and *thr* phenotypes are not identical since the DME cell bunching in late closure appeared to be unique to loss of *pim*. This suggested an additional role of *pim* that caused further defects in dorsal closure independent of the large cell defect.

#### Loss of the pair-rule genes odd-skipped, sloppy-paired 1, or paired causes isotropic lateral epidermal cell shapes:

**Figure 9:**
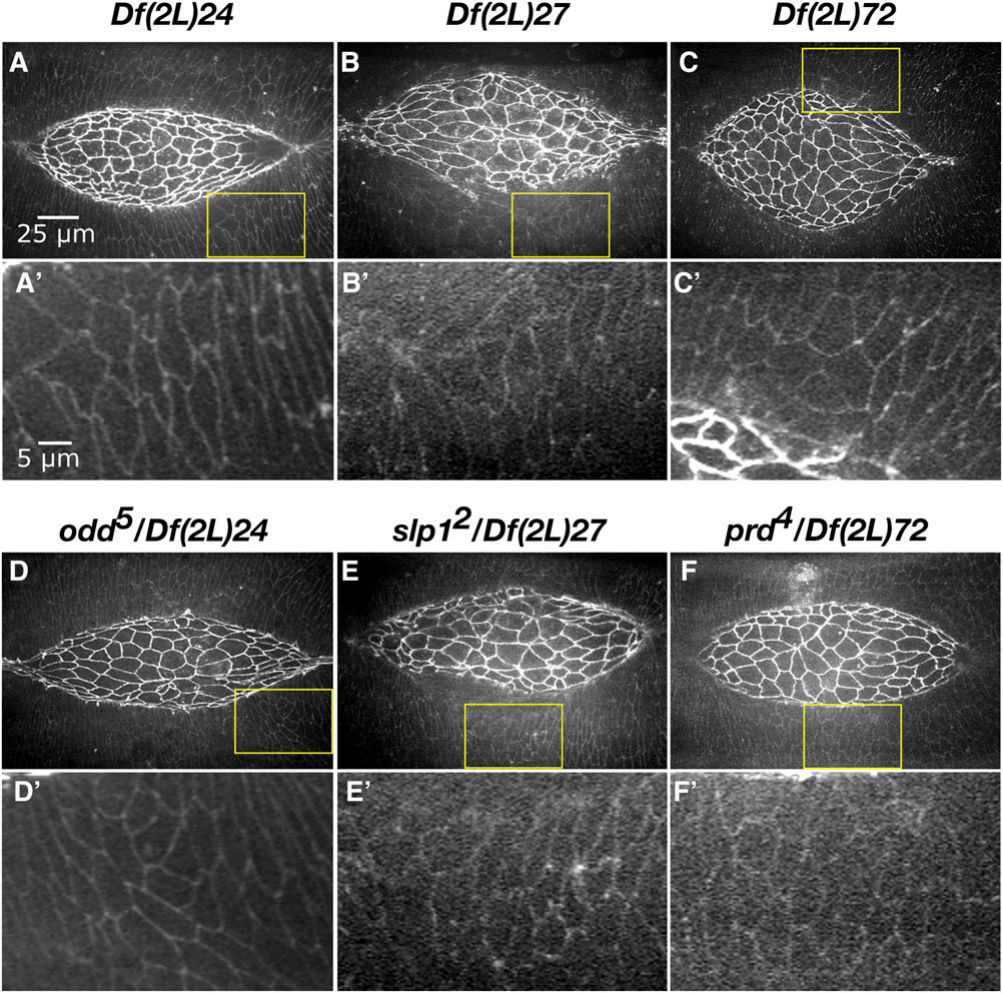
Pair-rule genes and the Dfs that remove them cause reduced elongation of lateral epidermal cells toward the dorsal midline (circumferentially along the dorsal-ventral axis). Stills from a time-lapse sequences of mid-dorsal closure stage, homozygous *Df(2L)24* (A), homozygous *Df(2L)27* (B) and homozygous *Df(2L)72* (C) embryos in a *D*E-cadherin-GFP imaging background. All have reduced elongation of lateral epidermal cells toward the dorsal midline (along the circumferential dorsal-ventral axis) and delete pair-rule genes. Transheterozygotes of pair-rule gene and Df partially phenocopy the reduced elongation of lateral epidermal cells in *odd*^5^ / *Df(2L)24* (D), *slp1*^*2*^ / *Df(2L)27* (E) and *prd*^*4*^* / Df(2L)72* (F) embryos. The yellow boxed areas in A, B, C, D, E and F are magnified in A’, B’, C’, D’, E’ and F’. Anterior is to the left, posterior to the right. The scale bar in A applies to A-F. The scale bar in A’ applies to A’ - F’. Stills are from Supp Movies 28-33, Supp Mov 23 provides an additional example of *Df(2L)27*.

The 2R Df screen identifies the loss of the pair-rule, patterning gene *even-skipped* (*eve*) in homozygous *Df(2R)16* embryos as the cause of isotropic lateral epidermal cells that do not elongate along the circumferential, dorsal-ventral axis. We found that four of the eleven 2L Dfs which had isotropic/unstretched lateral epidermal cell shapes also removed early patterning genes in the same pair-rule gene class as *eve*. *Df(2L)23* and *Df(2L)24* removed the pair-rule gene *odd-skipped* (*odd*) and live imaging of embryos homozygous for the Dfs showed reduced elongation of some of the lateral epidermal cells along the circumferential, dorsal-ventral axis. In addition, these Dfs had zipping defects. *Df(2L)23* had delayed formation of or was missing the posterior canthus and *Df(2L)24* developed a cigar-shaped opening in late closure. Transheterozygotes of *Df(2L)24* and *odd*^*5*^, a loss of function allele, similarly had reduced elongation of some of the lateral epidermal cells along the circumferential, dorsal-ventral axis; however, no penetrant zipping defects were observed (Figure 9A-A’). Thus, *odd* only partially accounted for the lateral epidermal phenotype of *Df(2L)24* and likely of *Df(2L)23*.

Embryos homozygous for *Df(2L)27* had isotropic lateral epidermal cell shapes. In five out of seven embryos imaged, the amnioserosa tore away from the leading edge. Three of the five homozygous *Df(2L)27* embryos did complete closure but had severe puckering and scarring of the lateral epidermal cells. *Df(2L)27* removed the pair rule genes *sloppy-paired 1* and *sloppy-paired 2* (*slp1* and *slp2*, respectively). Transheterozygotes of *Df(2L)27* and the *slp1* null allele, *slp1*^*2*^, had some lateral epidermal cells with reduced elongation along the circumferential, dorsal-ventral axis (Figure 9B-B’). This lateral epidermal phenotype was much milder than the phenotype observed in embryos homozygous for *Df(2L)27*. *slp1* and *slp2* can compensate for one another in segmental patterning (Cadigan *et al.* 1994), so we hypothesized that the milder lateral epidermal phenotype of transheterozygous *Df(2L)27* / *slp1*^*2*^ embryos may have been due to the presence of functional Slp2 protein. Further testing with *slp1* and *slp2* double mutants will be necessary to determine whether loss of these two genes can account completely for the severe lateral epidermal phenotype of *Df(2L)27*.

The final 2L Df that removes a pair rule gene, *Df(2L)72*, had reduced elongation of some lateral epidermal cells, primarily in the posterior half of the embryo (five of the seven embryos imaged). Additionally, four of these embryos also developed a very rounded dorsal opening, likely due to zipping defects. *paired* (*prd*) is removed by *Df(2L)72* and transheterozygotes of *Df(2L)72* and *prd*^*4*^, a null allele, phenocopied the lateral epidermal phenotype of homozygous *Df(2L)72* embryos, but not the rounded dorsal opening (Figure 9C-C’). Thus, an additional gene removed by *Df(2L)72* must have been responsible for the zipping defect. Interestingly, *Df(2L)46* and *47* removed the known dorsal closure gene *wg*, which is a segment polarity gene and a downstream effector of pair-rule genes (Ingham *et al.* 1988). Previous studies of dorsal closure in the absence of *wg* have documented a loss of lateral epidermal cell elongation toward the dorsal midline (along the circumferential, dorsal-ventral axis) similar to the phenotype observed in *Df(2L)46* and *47* (McEwen *et al.* 2000; Kaltschmidt *et al.* 2002; Morel and Martinez Arias 2004). Thus, over half of the Dfs that caused isotropic/unstretched lateral epidermal cell shapes were due to early patterning genes and their regulation of Wg signaling, suggesting that early patterning provides transcriptional cues for the elongation of the lateral epidermal cells along the circumferential, dorsal-ventral axis. We speculate that this may be due to roles in microtubule orientation or in upstream regulation of cell polarity.

## Conclusions

**Figure 10:**
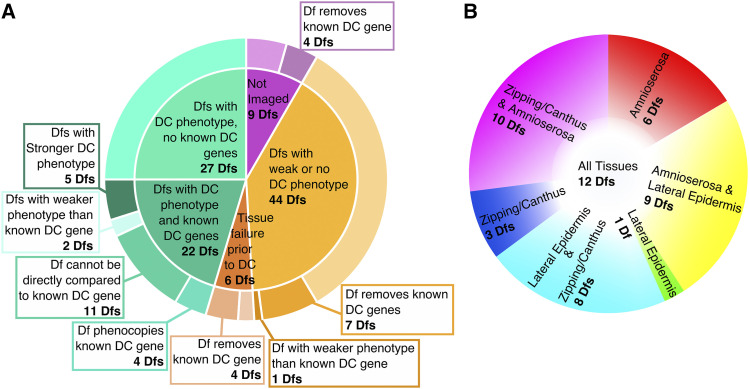
Df 2L screen summarized in pie charts by the number of Dfs causing a particular dorsal closure phenotype, and the tissues that are affected. Forty-nine of the 108 Dfs in the 2L Df kit have a penetrant, mid to severe dorsal closure phenotype, 27 of which do not remove a known dorsal closure gene (A). Some Dfs remove a known dorsal closure gene and are more severe than the published phenotype of the gene, therefore an additional, new dorsal closure gene is likely deleted in the interval. The 49 Dfs with a dorsal closure phenotype affect one or more tissues or processes (B). The color coding of phenotype categories in B corresponds to Appendix A column 1.

We have successfully screened 2,411 genes of the 2,765 genes on 2L (87.2%) and identified 49 genetic regions to have notable, diverse defects in closure (summarized in Figure 10). Of these, 27 Dfs did not remove a gene known to affect dorsal closure. Phenotypes included defects in cell shape, canthus formation, and tissue dynamics. To date we have identified four and possibly five, new genes affecting dorsal closure, *pim*, *odd*, *prd*, and *slp1* (and likely *slp2*) on 2L based on Df dorsal closure phenotypes similar to those identified on 2R. We anticipate that further analysis of these 2L deficiencies will lead to the identification of several new and novel dorsal closure genes. Consequently, we expect to identify links between pathways and structures already known to coordinate various aspects of closure as well as new processes and pathways that contribute to closure.

Embryos homozygous for *pim*, *odd*, *prd*, and *slp1* started dorsal closure with defects such as large, disorganized, and/or isotropic lateral epidermal cell shapes, suggesting processes prior to dorsal closure are disrupted. We classify these genes, as well as the 18 Dfs on 2L that start dorsal closure with irregularities, as “pre-dorsal closure” genes and Dfs. While the genes responsible for the pre-dorsal closure Dfs may not be actively contributing to dorsal closure, the phenotypes provide valuable insight into the robust nature of this process. These pre-dorsal closure genes and Dfs demonstrate how prior development sets the stage for successful closure, but also emphasize that such defects may not necessarily prevent it from proceeding to completion. For example, how the lack of elongation of the DME cells toward the dorsal midline influences the efficiency of zipping and adhesion between the DME cells and the PAS cells is an open question. Investigation of DME cell identity in these pre-dorsal closure genes affecting the lateral epidermis will be useful in establishing whether they feed into well studied pathways important for lateral epidermal cell morphology and behavior such as JNK and Wnt signaling or are due to a novel pathway that is upstream or in tandem with these key pathways (McEwen *et al.* 2000; Kaltschmidt *et al.* 2002; Morel and Martinez Arias 2004; Fernández *et al.* 2007; Chatterjee and Bohmann 2012; Lada *et al.* 2012).

Thirty-nine of the 49 Dfs with significant morphological defects in dorsal closure completed dorsal closure. As one might expect with such a diverse set of defects, the time required for closure to complete was highly variable – some Dfs completed dorsal closure within the same time span of controls (approximately three to four hours) while the most delayed Dfs took over seven hours to complete. Because we do not know if the delay in dorsal closure is due to the morphological defects of the dorsal closure gene(s) removed or an added affect from other genes within the Df, further characterization of the rate of closure awaits identification of the gene or genes responsible for the morphological defects in closure.

We anticipated that some Dfs would remove genes necessary for developmental stages prior to dorsal closure and that we would therefore be unable to assess dorsal closure in such homozygous Df embryos. Indeed, six of the 99 2L Dfs imaged did not progress to dorsal closure stages due to severe tissue failure in early embryogenesis. In contrast, when we completed the 2R Df screen we were very surprised that all 88 2R Dfs that we were able to cross into the imaging line background made it to dorsal closure (Mortensen *et al.* 2018). Maternally loaded protein and/or RNA products of all essential early embryonic genes was apparently enough to get the 88 2R Dfs to dorsal closure stages. Additionally, overexpression of *D*E-cadherin from the imaging transgene may have rescued some adhesion defects. This effect was the case in *Df(2R)72* which removes *shotgun*, the gene encoding *D*E-cadherin (Mortensen *et al.* 2018). This raises two important features of our screen that need to be considered. First, genes whose products are important for dorsal closure, but are maternally loaded and perdure through dorsal closure stages will not be detected. Second, overexpression of *D*E-cadherin by the ubiquitously driven *D*E-cadherin-GFP imaging line can mask adhesion defects that might be penetrant in other imaging backgrounds (detailed in Mortensen *et al.* 2018). Therefore, our screen might miss key genes important for adhesion that interact with *D*E-cadherin. This may explain the reduced severity of *Df(2L)42*, *43* and *78* in comparison to the known dorsal closure genes they remove. Since *shotgun*, which encodes *D*E-cadherin, resides on the 2R, we may be able to ameliorate this issue in future Df screens of genes on the X, 3L, 3R, and 4^th^ chromosomes by using an endogenously labeled *D*E-cadherin imaging line (Huang *et al.* 2009).

With the added experience of this screen of 2L, we identified additional sub-classes of phenotypic characterizations that have provided better insight into the observed defects. For example, we find that embryos that are homozygous for Dfs that cause the amnioserosa to fall apart are either due to increased ingressions in the central amnioserosa or a breakdown of the adherens junctions – both lead to loss of cell-cell adhesion within the tissue, but likely through distinct, molecular mechanisms. By parsing out these patterns within a category we can learn which changes in cellular function lead to an observed defect in closure and further inform the search for the candidate gene(s) responsible for the phenotype of the Df. In addition, we identified one new phenotype category, increased asymmetry of zipping, that we did not observe on 2R – uncovering yet another way in which the tissues and process of dorsal closure can be perturbed.

With the analysis of 2R, published previously (Mortensen *et al.* 2018) and now the 2L, we have screened 5,416 genes (>92.5%) of the 5,854 genes on the 2^nd^ chromosome for dorsal closure defects. We have identified 96 Dfs with a penetrant, strong to mid-severity closure phenotype, 45 of which do not remove a known dorsal closure gene. An additional 17 Dfs have more severe dorsal closure phenotypes than that published for the known dorsal closure gene the Df deletes, suggesting that another gene which contributes to closure is removed by these Dfs. Additionally, the dissection of the complex dorsal closure phenotype of *Df(2L)56* using overlapping sub-Dfs exemplifies how genetic dissection of the Df can identify sub-regions responsible for multiple, genetically distinct phenotypes (Figure 5). Because of the complex Df phenotypes we observe and the possibility that some dorsal closure Dfs may delete more than one dorsal closure gene, we expect to uncover more than 62 novel dorsal closure genes on the second chromosome.

The intertwining of phenotypes demonstrates the emergent properties of dorsal closure. Furthermore, the diversity of phenotypes observed in the Df screen of the second chromosome, and the ability to complete closure in spite of them, further demonstrates the robust and resilient nature of this process. The richness of phenotypes that cannot be explained by previously published dorsal closure genes suggests that there is still much to be discovered and understood about epithelial sheet morphogenesis.
